# Epigenetically mismatched parental centromeres trigger genome elimination in hybrids

**DOI:** 10.1126/sciadv.abk1151

**Published:** 2021-11-19

**Authors:** Mohan P. A. Marimuthu, Ravi Maruthachalam, Ramesh Bondada, Sundaram Kuppu, Ek Han Tan, Anne Britt, Simon W. L. Chan, Luca Comai

**Affiliations:** 1 UC Davis Genome Center, UC Davis, Davis, CA, USA.; 2 Department of Plant Biology, UC Davis, Davis, CA, USA.; 3 School of Biology, Indian Institute of Science Education and Research (IISER), Thiruvananthapuram, Vithura, Kerala 695551, India.; 4 University of Maine, Orono, ME, USA.

## Abstract

Wide crosses result in postzygotic elimination of one parental chromosome set, but the mechanisms that result in such differential fate are poorly understood. Here, we show that alterations of centromeric histone H3 (CENH3) lead to its selective removal from centromeres of mature *Arabidopsis* eggs and early zygotes, while wild-type CENH3 persists. In the hybrid zygotes and embryos, CENH3 and essential centromere proteins load preferentially on the CENH3-rich centromeres of the wild-type parent, while CENH3-depleted centromeres fail to reconstitute new CENH3-chromatin and the kinetochore and are frequently lost. Genome elimination is opposed by E3 ubiquitin ligase VIM1. We propose a model based on cooperative binding of CENH3 to chromatin to explain the differential CENH3 loading rates. Thus, parental CENH3 polymorphisms result in epigenetically distinct centromeres that instantiate a strong mating barrier and produce haploids.

## Teaser

CENH3 variation mediates parental centromere imbalance.

## INTRODUCTION

**Fig. 1.**
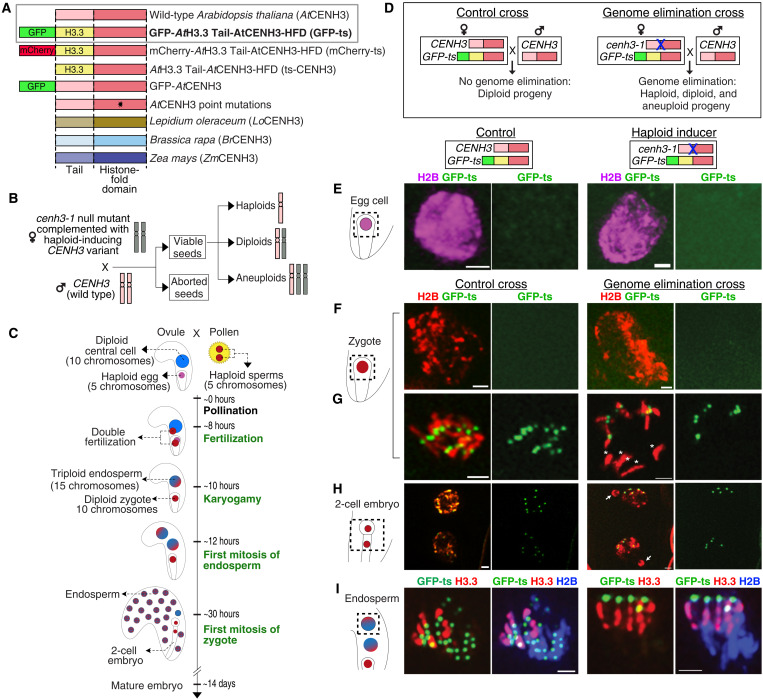
Biased localization of GFP-ts in zygotes, early embryos, and endosperm from GE crosses in *Arabidopsis*. (**A**) Structure of wild-type AtCENH3, GFP-ts (gray box), and other haploid-inducing CENH3 variants. All CENH3 variants are expressed under the control of the *Arabidopsis CENH3* regulatory sequences and, in the *cenh3-1* null mutant background, act as HIs. GFP-ts is highlighted in bold because of its extensive use in this study: It results in the highest haploid induction rate, and it labels the centromeres efficiently. (**B**) CENH3-mediated GE in *Arabidopsis*. (**C**) Progressive stages of fertilization and early seed development. Landmark events described in this study are highlighted in green. (**D**) The GFP-ts is expressed maternally in both the control cross (CC) and the GE cross (GEC). However, in the GEC, the maternal line is homozygous for the *cenh3-1* mutation. (**E** to **I**) The ovule schematic on the left indicates the region of interest in the ovule shown on the right. (E) GFP-ts is absent from the egg nucleus before fertilization and (F) in the early zygote. (G) GFP-ts reappears before mitosis on the centromeres of normally segregating chromosomes in both CC and GEC. In GEC, GFP-ts is absent on five chromosomes “*,” which lag and (H) form micronuclei in two-cell embryos (arrows). (I) Metaphase (CC) and anaphase (GEC) endosperm chromosomes displaying male-only (red) and biparental (blue) chromatin. Note the paternal bias in loading of GFP-ts in GEC. Scale bars, 1 μm.

Uniparental genome elimination (GE) entails the postzygotic loss of one parental chromosome set. Distant hybridization can result in GE (*1*), but notwithstanding their basic scientific interest and their usefulness in producing haploids for breeding, little is known about what mechanisms mediate identification and selective missegregation of one parental chromosome set. In *Arabidopsis* (*2*), maize (*3*), and wheat (*4*), manipulation of centromeric histone H3 (*CENH3*) results in efficient GE in isogenic crosses. CENH3, aka CENP-A, is an essential histone H3 variant that determines centromere identity by forming a specialized chromatin on which the kinetochore assembles (*5*). When a *cenh3* embryo-lethal mutant in *Arabidopsis* is complemented by a haploid inducer (HI) CENH3 variant (Fig. 1A), selfing has no effect on seed set or genome maintenance (*2*, *6*–*8*), but outcrossing to the wild-type (WT) male results in ~70% seed death. Of the viable seeds, up to 40% are haploid; the rest are diploid and aneuploid in roughly equal ratio (Fig. 1B). Thus, outcrossing to an isogenic wild type yields a strongly incompatible outcome triggering GE, suggesting a role for a differential epigenetic mark in establishing a hybridization barrier. Here, we leverage the *Arabidopsis* system to understand the molecular and cytological basis of GE. We show that variant CENH3s are selectively removed from centromeres during reproduction, while wild-type CENH3 is retained. In the hybrid embryos, CENH3 and the kinetochore assemble on the CENH3-rich centromeres inherited from the wild-type parent, but not on those from the HI. Frequently, the HI chromosomes missegregate, form micronuclei, and are lost. Alternatively, the HI centromeres can gradually regain strength, a process favored by the E3 ubiquitin ligase VARIANT IN METHYLATION 1 (VIM1). We propose a model to explain the differential CENH3 loading rates by a cooperative binding effect.

## RESULTS

### Biased loading of a CENH3 variant in zygote precedes GE

We searched for signs of GE in zygotes and early embryonic mitoses (Fig. 1, C and E to I, and fig. S1) (*9*–*12*) by tracking female and male chromatin distinctly labeled with histone H2B fusion tags (Fig. 1C and Materials and Methods). Among the HIs (Fig. 1A), the green fluorescent protein-tailswap (GFP-ts) variant is highly efficient in triggering GE when crossed to WT male (Fig. 1B). In addition, the GFP-ts fluorescently marks the centromeres for easy visualization. Hence, we used *cenh3-1*;*GFP-ts* X *WT* cross (Fig. 1D) as a representative GE cross (GEC) throughout the study. Lines coexpressing endogenous CENH3 and GFP-ts behave as wild type (*2*), yielding only diploid progeny on crossing to wild-type male and thus were used as an isogenic control cross (CC) (Fig. 1D). Reconfirming previous reports (*13*), centromeric GFP-ts signals were absent in haploid egg cells (Fig. 1E) of both control and HI lines. We used WT males expressing H2B-tdTomato (*14*) to identify zygotic chromatin. Following fertilization, centromeric GFP-ts signals were still absent in zygotes of both CC and GEC until 19 hours after pollination (HAP) (Fig. 1F). As the zygotic mitosis progressed (20 to 36 HAP), the GFP-ts appeared on all 10 centromeres in the CC. In contrast, GEC displayed only five signals [Fig. 1G and fig. S1, A (b) and B (b)], consistent with uniparental loading. The 10 versus 5 patterns of GFP-ts persisted throughout embryonic mitoses in both CC (100%; *n* = 38) and GEC (86.4%; *n* = 125) (Fig. 1H and figs. S1, A and B, and S2K). The CC embryos showed normal chromosome segregation, whereas GEC displayed laggards and micronuclei-lacking centromeric GFP-ts during zygotic anaphase and telophase [Fig. 1H and fig. S1, A (d) and B (d)]. Thus, while both CC and GEC displayed zygotic reprogramming (*13*), GFP-ts reloaded only on the five centromeres associated with properly segregating chromosomes in GEC.

### Biased loading of GFP-ts on wild-type centromeres in early GEC endosperm

Higher seed death in the haploid induction cross could be explained by endosperm failure (*15*–*17*) probably hastened by missegregation of HI chromosomes (*17*). To test this hypothesis, we examined the behavior of HI chromosomes in the endosperm by marking parental chromatin with fluorescent tags (Fig. 1C). Triploid endosperm (10 maternal + 5 paternal) is the second product of double fertilization nourishing developing embryos (Fig. 1C). Following central cell fertilization, the resulting endosperm proliferates rapidly (from ~11 HAP), whereas the zygote takes ~30 HAP for its first division (*18*). As in egg cells, centromeric GFP-ts signals were absent in the central cell nuclei before fertilization in both control and HI lines (see below, Fig. 6, F and G). Here, we used WT males expressing red fluorescent protein (RFP)–tagged, sperm-specific histone H3.3 variant (HTR10) (*19*) for investigating very early stages of endosperm development. After fertilization, paternal chromatin was still marked by HTR10-RFP, which is pronounced on endosperm chromatin (figs. S1, D and F, and S2, C, F, and G). After karyogamy, maternal GFP-ts was loaded rapidly on all 15 parental centromeres (84%, *n* = 94; figs. S1C and S2B) in CC and only on 5 in the GEC instead (90%, *n* = 27; figs. S1E and S2, F and G). In GEC, all five centromeric GFP-ts signals were predominantly associated with male chromatin (HTR10-RFP) and were absent from the chromatin inherited from the female (Fig. 1I and figs. S1F and S2G). As HTR10-RFP fluorescence diminished after the second mitosis (*19*), we used *pRPS5A-H2B-tdtomato* (*14*) as a male chromatin marker for examining later stages. In subsequent endosperm mitoses (20 to 36 HAP) of the GEC, in addition to nuclei with 5 GFP-ts signals, we often detected nuclei with 10 or 20 brighter signals or a variable number of bright and faint signals (fig. S2, H and I). Numbers that are multiples of five are consistent with nuclear restitution. In contrast, up to 15 signals were observed consistently in the CC (fig. S2, D and E). Suggestive of genome instability, rare chromosome bridges (fig. S1F) and micronuclei (fig. S2I) were found in the endosperm of GEC.

GFP-ts reloading patterns in both the embryo and endosperm (fig. S2, J and K) confirmed the zygotic reprogramming of CENH3 (*13*), except for biased loading in GEC. GFP-ts localization in GEC to five centromeres, the gametic number, was consistent with uniparental bias and failure to reload on the centromeres inherited from the HI parent.

### Interploidy GECs confirm depletion of GFP-ts from HI parent chromosomes

**Fig. 2.**
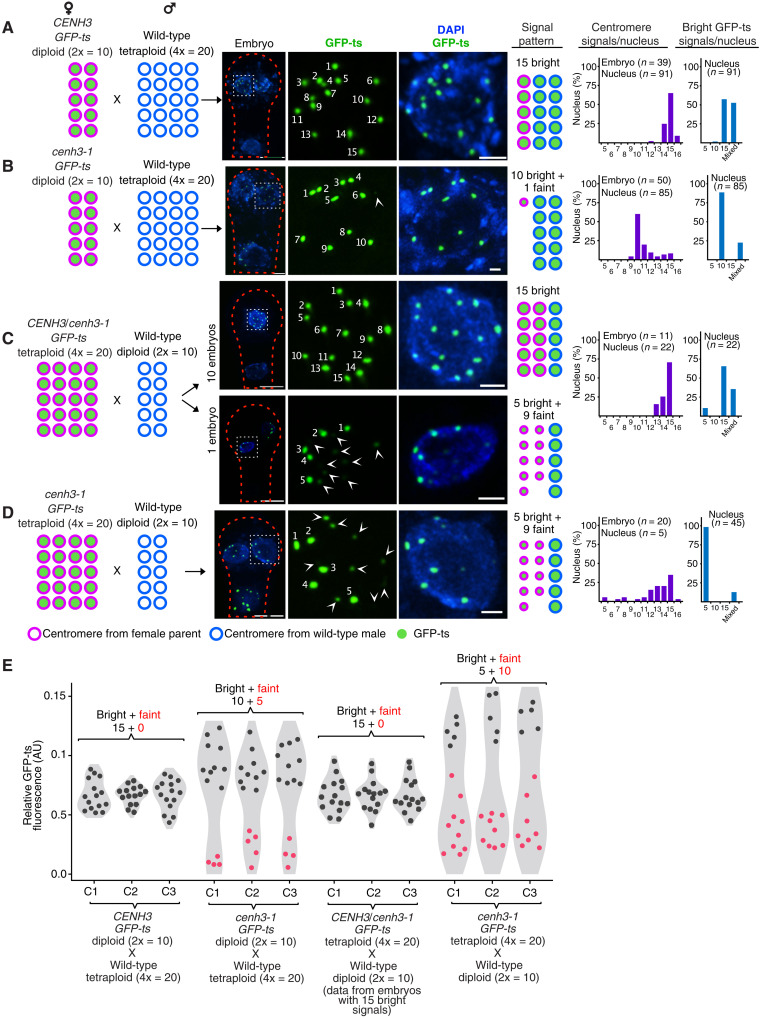
Interploidy GECs confirm depletion of GFP-ts from HI parent chromosomes. Tetraploid (**A** and **B**) and diploid (**C** and **D**) *Arabidopsis* wild-type strains were crossed as males with diploid (A and B) or tetraploid (C and D) females expressing GFP-ts in *CENH3^+/+^* (A) *cenh3-1^−/−^* (B and D) *CENH3^+/−^* (C) backgrounds. A selected nucleus (white dotted box) on the embryo is shown enlarged on the right (A to D). Arrowheads mark the faint GFP-ts signals. (**E**) Relative fluorescence intensity of centromeric GFP-ts signals. Fainter signals from the GECs are highlighted in red circles. Each column represents normalized intensity in arbitrary units (AU) within a single cell (C1 to C3) for each cross. Scale bars, 5 μm (for the embryo images) and 1 μm (for the individual nucleus images). DAPI, 4′,6-diamidino-2-phenylindole.

The above data suggested that centromeres inherited from one parent, presumably the HI, are incompetent for CENH3 loading. To test this hypothesis, we tracked the genome of origin using parents that differed in ploidy. Interploidy crosses, such as 4x(tetraploid) X 2x(diploid) and the reciprocal cross, are possible in *Arabidopsis* (*17*, *20*–*22*). In the triploid (3x) embryos produced by an interploidy CC [2x *CENH3;GFP-ts* X 4x WT], a majority of nuclei exhibited the expected 15 centromeric GFP-ts signals (Fig. 2A). Whereas only 10 bright signals, with an additional 5 or fewer faint signals were seen in 3x embryos from the (2x *cenh3-1;GFP-ts* X 4x WT) GEC (Fig. 2B). This pattern is possible only if the maternal GFP-ts is loaded to the wild-type paternal centromeres. Conversely, in 3x embryos from the reciprocal ploidy cross [4x *CENH3(+/−);GFP-ts* X 2x WT], 10 of 11 embryos exhibited the expected 15 signal/nucleus, except for one embryo, in which every nucleus showed five bright signals along with 9 or 10 fainter signals (Fig. 2C). This HI-like behavior could be associated with maternal inheritance of two null *cenh3-1* alleles, a hypothesis verified below (Fig. 7, A and B). Furthermore, in 3x embryos from the GEC (4x *cenh3-1;GFPts* X 2x WT) (Fig. 2D), most nuclei exhibited five brighter signals, along with 7 to 11 fainter signals. Together, we concluded that, in the GEC, maternally inherited GFP-ts displayed biased localization on the centromeres inherited from the wild-type parent (Fig. 2E). Faint centromeric GFP-ts signals in postzygotic cell stages were consistent with progressive reloading of kinetochore components on revenant maternal centromeres (see below, Fig. 8A).

### HI centromeres sustain partial centromeric identity

**Fig. 3.**
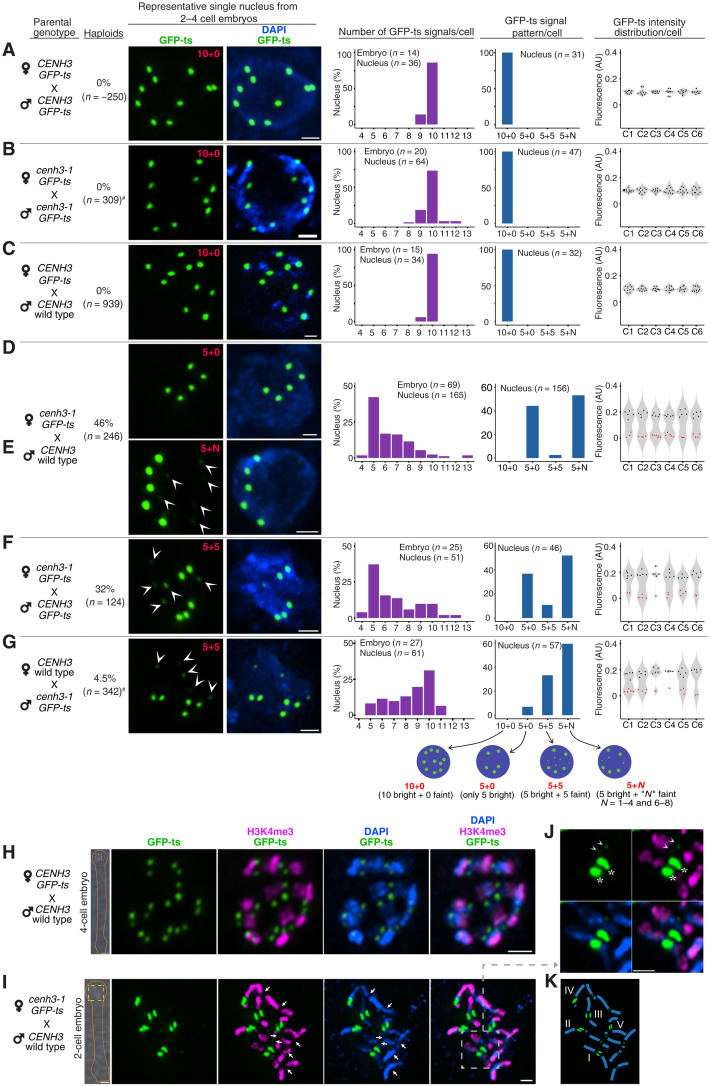
Uniparental loading of GFP-ts on centromeres of two– to four–cell stage embryos during GE. (**A** to **C**) GFP-ts forms 10 strong centromeric signals in early embryonic nuclei in a set of CCs, while only five strong signals are visible in GECs (**D** to **G**). In some cases, up to six weak signals (arrowheads) are also visible (E to G), indicating suboptimal loading onto one parental centromere set. “*n*,” number of nuclei or embryos scored. The green channel is enhanced to reveal the fainter signals (E to G). For the displayed images (A to G), corresponding patterns of GFP-ts signals are marked on the green channel for each genotype. Bar graphs and violin plots display the number and pattern of GFP-ts signals per cell, respectively, and relative fluorescence intensity in AU for the corresponding genotypes is shown on the left. Each column of the violin plots displays relative GFP-ts intensity within a single cell (C1 to C6) with fainter GFP-ts signals highlighted in red circles. (**H** and **I**) Whole embryos are shown on the left side, and the highlighted nuclei with the yellow dotted line are shown on the right. (H) Prometaphase cells of the CC displaying 10 pairs of signals. In comparison, the top cell from the GEC (I) displayed five pairs of bright signals along with faint signals (**J**) (enhanced to display faint GFP-ts signals) on multiple chromosomes. Arrows mark maternal chromosomes; arrowheads indicate faint GFP-ts signals; asterisk (*) indicates bright signals. (**K**) Inferred WT karyotype and location of GFP-ts signals of the marqueed nucleus in (I). The single nuclei data shown in (A) to (I) were obtained from whole-embryo images shown in fig. S3 (C to J). Note that the embryos shown in (H) and (I) are enlarged for clarity in fig. S3 (J and K, respectively). #Data from (*2*, *39*). Scale bars, 1 μm (for single nucleus) and 5 μm (for whole-embryo images).

If centromeres in the HI female gametes (Figs. 1 and 2) lose their identity in an outcross, how do they maintain stability during self-pollination? We hypothesized that loss of identity is partial since haploid yield is below 50%. To document this, we examined centromeres in self-pollinated two- to four-cell embryos of the control (*CENH3;GFP-ts*; Fig. 3A and fig. S3C), HI (*cenh3-1;GFP-ts*; Fig. 3B and fig. S3D), WT (fig. S3, A and I), and *cenh3-1;GFP-CENH3* (fig. S3, B and J) lines and in the CC (Fig. 3C and fig. S3E). In all types, the centromeric signals’ modal value was 10 per cell. Similarly, embryos from these genotypes displayed comparable variation in centromere signal intensity (1.2- to 2.5-fold difference; violin plots in Fig. 3 and fig. S3) without distinct patterns as observed in Fig. 2E.

In contrast, embryos from the GEC displayed five bright centromeric GFP-ts signals along with one to eight fainter GFP-ts signals (*n* = 165 nuclei, 69 embryos; Fig. 3, D and E, and fig. S3F). Within each nucleus, the brightest signal was 14- to 34-fold stronger than the weakest signal (violin plots), forming three recognizable patterns (bright+faint): 5+0 (44%), 5+5 (3%), and 5+*N* (53%, where *N* = 1 to 4 and 6 to 8) in contrast to 10+0 in all controls. Furthermore, 28% of embryos displayed only the 5+0 pattern in all cells, whereas the rest displayed combinations of all three patterns. Even the biparental provision of GFP-ts (*cenh3-1;GFP-ts* X *CENH3;GFP-ts*) did not alter the GFP signal patterns, which remained similar to *cenh3-1;GFP-ts* X *WT* (Fig. 3F and fig. S3G). Similarly, a paternal mCherry tag (*cenh3-1;GFP-ts* X *CENH3;mCherry-ts*) did not alter the outcome: Five bright GFP-ts signals as in *cenh3-1;GFP-ts* X *CENH3:GFPts* were colocalized with bright mCherry-ts signals (fig. S3M). We further confirmed the uniparental localization of GFP-ts by comparing cells arrested in the embryonic prometaphase stage (see Materials and Methods) from the CC (Fig. 3H and fig. S3K) and GEC (Fig. 3I and fig. S3L). As inferred from the derived karyotype of the highlighted GEC cell (Fig. 3, I and K), GFP-ts strongly marked one of the the WT parent centromere set. The same cell also displayed missegregated chromosomes (up to seven) from the first zygotic mitosis that presented very faint centromeric GFP-ts signals (Fig. 3J). Together, the presence of >5 or <5 faint signals and 5+5, 5+*N* patterns of GFP-ts within a nucleus were consistent with chromosome missegregation and gradual centromeric reloading of GFP-ts during early embryo development.

When crossed as males, most HI types yield no haploids. The only exception is the strongest HI, GFP-ts. When used in the male GEC (WT X *cenh3-1;GFP-ts*), it generates ~4% haploids, 10-fold fewer than the normal GEC (*2*). In the male GEC, nuclei with only five bright centromere signals were less common: 7% 5+0 pattern versus 44% for *GFP-ts* X WT (*P* < 0.0001, two-sample *z* test), suggesting that diminished removal from HI centromeres resulted in improved reloading and caused lower GE (Fig. 3G and fig. S3H; see fig. S6B(a).

Our analysis revealed the following properties: (i) Selfing the HI strain mimics the wild-type behavior, resulting in proper reloading of GFP-ts on all centromeres despite its prior removal. (ii) Cytological analyses reconfirm biased GFP-ts occupancy of centromeres inherited from one parent (Fig. 3, I to K). (iii) Male-inherited HI centromeres are reloaded more efficiently, explaining the lower haploid yield from male HI. These observations suggest that competitive centromeric reloading in the GEC occurs because of the strong previous depletion of the variant CENH3, and not its presence, on the HI gametic centromere set.

### In GECs, wild-type and variant CENH3s localize to centromeres contributed by the wild-type parents

**Fig. 4.**
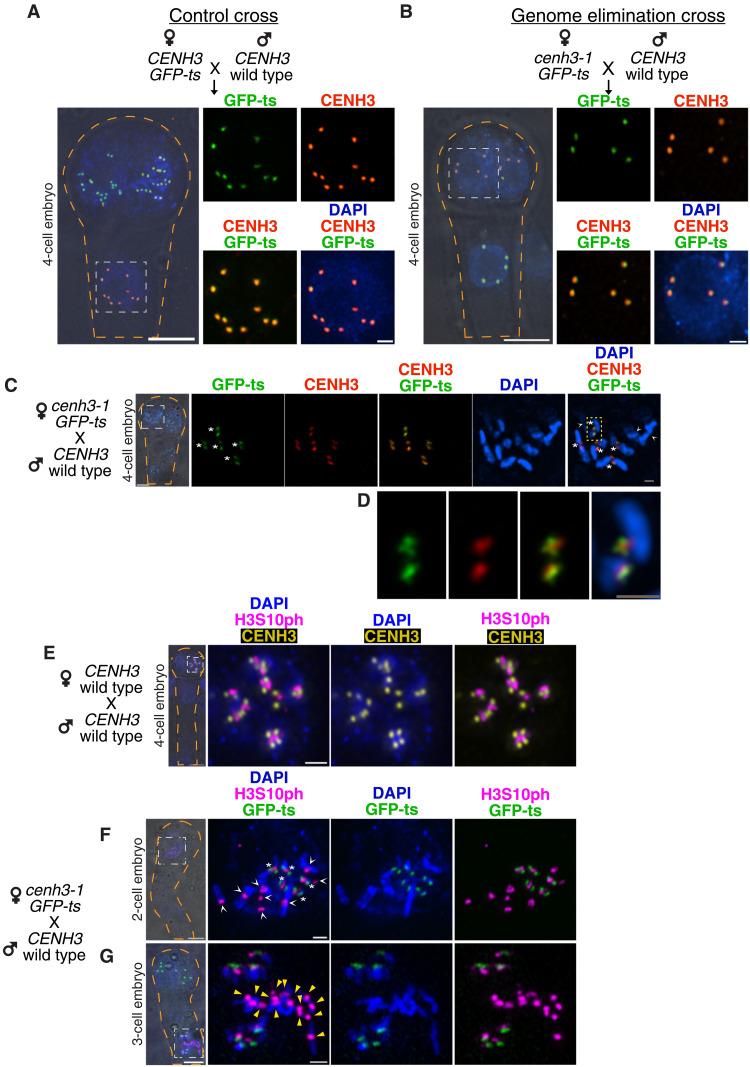
WT CENH3 and GFP-ts occupy the same functional centromeres during haploid induction. (**A** to **G**) A selected nucleus (white dotted box) on the embryo is shown enlarged on the right. (A) Colocalization of CENH3 and GFP-ts on 10 centromeres in the CC. (B) Colocalization on five centromeres in the GEC. (C) 3D-SIM image of prometaphase stage cells in embryos from the GEC. (D) Enlarged condensed chromosome from image (C) (yellow dotted box). (E) At the pericentromeric region of all chromosomes, H3S10ph (phosphorylation of H3 at serine-10) is noticeable as an independent domain in prometaphase stage nuclei in the WT embryo and in prometaphase (F) and anaphase (G) in the embryo from the GEC. Asterisk (*) marks wild-type centromeres, whereas the arrowheads mark the chromosomes lacking GFP-ts. Yellow triangles (G) mark laggard chromosomes with H3S10ph signals, but lacking GFP-ts. Scale bars, 5 μm (for the whole-embryo images) and 1 μm (for the rest of the figure).

Next, we investigated the fate of paternally contributed WT-CENH3 by immunodetection (*23*), leveraging the absence of CENH3 N terminus antigens on GFP-ts and GFP antigens on WT-CENH3 (Fig. 1A and fig. S4A). In the CC, both GFP-ts and WT-CENH3 variants colocalized on all 10 centromeres (Fig. 4A). In the GEC, both variants colocalized on five centromeres inherited from the wild-type parent in interphase (Fig. 4B), G_2_ (fig. S4B), prometaphase (Fig. 4C), and anaphase cells (fig. S4C). Often, faint GFP-ts signals colocalized with faint WT-CENH3 signals (fig. S4C). In addition, both CENH3 and GFP-ts domains partially overlapped [three-dimensional structured illumination microscopy (3D-SIM); Fig. 4, C and D], suggesting the formation of centromeric subdomains enriched with one or the other protein, consistent with previous observations with natural or artificial combinations of CENH3 types (*12*, *24*, *25*). We concluded that native or GE-inducing CENH3s display similar localization bias in the GEC, indicating that only the wild-type centromeres are competent during the very early embryonic mitosis.

### In GECs, typical centromeric chromatin states persist on defective chromosomes

We wondered whether the loss of CENH3 during GE affects the centromere and pericentromeric stereotypical chromatin states. Phosphorylation of H3 at serine-10 (H3S10ph) is found in plants on pericentric chromatin of condensed chromosomes (*26*–*29*). On the other hand, H3K4me3 marks the euchromatic region but is excluded from the centromere proper (*30*). In the prometaphase cell from WT embryos, the H3S10ph signal marks a domain at the pericentric region that is spatially distinct from the CENH3 signals and labeled all 10 chromosomes (Fig. 4E). Similar to the wild type, in the prometaphase cell from the GEC embryos, chromosomes inherited from both parents displayed H3S10ph signals, but only wild-type chromosomes had GFP-ts signals (Fig. 4F). Even after sister chromatid cohesion resolved at anaphase, we found H3S10ph on leading sister chromatids with functional centromeres, as well as on the lagging chromatids, which lack GFP-ts (Fig. 4G). The euchromatin-specific H3K4me3 was excluded in the pericentric and centromeric region on chromosomes inherited from both wild-type and GFP-ts parents but strongly stained the euchromatic arms (Fig. 3, H and I). The H3K9me2, a heterochromatic mark (*31*), was also found on both leading and lagging chromatids (fig. S7B). In conclusion, because normal centromeric patterns of histone H3 modifications persisted after differential CENH3 loading and during centromeric failure, they are unlikely to underlie chromosome missegregation and loss.

### HI chromosomes assembled defective kinetochores in GEC

**Fig. 5.**
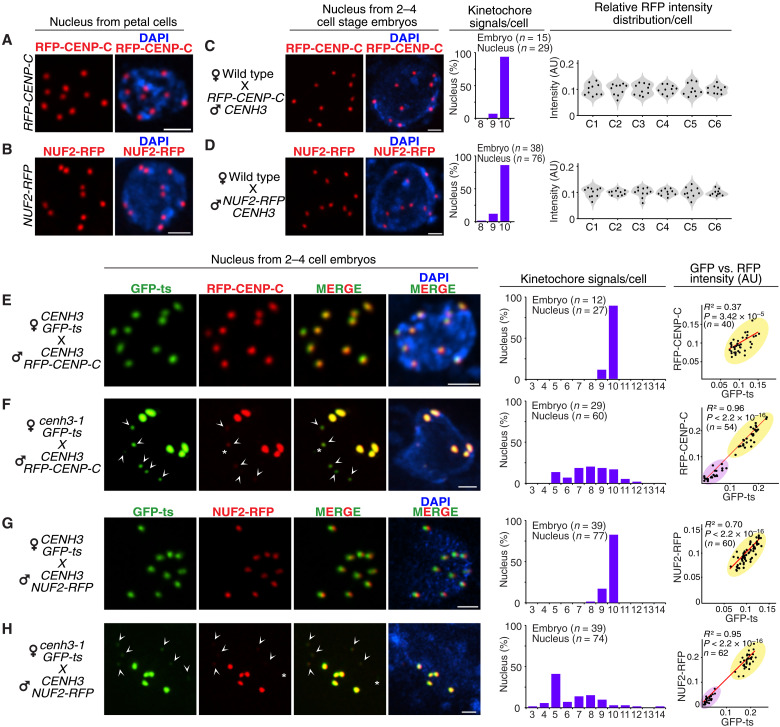
Uniparental assembly of functional kinetochores in embryos undergoing GE. (**A** and **B**) Localization of RFP-tagged CENP-C and NUF2 in an interphase nucleus from petals. (**C** and **D**) Localization of paternal RFP-tagged CENP-C (C) and NUF2 (D) on all 10 parental kinetochores in the interphase nuclei of two– to four–cell stage embryos from a CC using WT females. (**E** to **H**) Comparison of GFP-ts, CENP-C, and NUF2 colocalization in interphase nuclei from two– to four–cell stage embryos in the CC (E and G) and GEC (F and H). White arrowheads and asterisks (*) in (F) and (H) mark faint signals and singleton GFP or RFP signals, respectively. Bar graphs, violin plots, and correlation plots represent the kinetochore numbers and relative fluorescent intensity values (AU) for the genotypes shown on the left. Each column of the violin plots indicates relative RFP intensity within a single cell (C1 to C6). Scale bars, 1 μm.

If biased loading of CENH3 and GFP-ts causes uniparental centromere dysfunction, then this outcome should be reflected in a kinetochore defect. To demonstrate this, we examined RFP-tagged CENP-C and NUF2, essential components of inner and outer kinetochore protein complexes, respectively (*32*). Marking the kinetochores, both fusion proteins produced 10 centromeric fluorescent signals in somatic nuclei (Fig. 5, A and B). When paternally contributed in CCs, both marked all 10 parental centromeres equally in two– to four–cell stage embryos (wild-type female; Fig. 5, C and D) and colocalized with maternal GFP-ts (*CENH3;GFP-ts* female; Fig. 5, E and G) and matched it in intensity. Whereas in GECs, both RFP–CENP-C and NUF2-RFP signals (Fig. 5, F and H) presented wide numerical variation and bright+faint patterns similar to GFP-ts. In both GEC cases, bright RFP signals colocalized with the bright GFP-ts signals. Singleton faint signals were rarely observed (Fig. 5, F and H, asterisk marks). We concluded that, in GEC only, the wild-type parental chromosome set assembled optimal kinetochores while, on the HI set, kinetochores were either suboptimal or absent.

### Chromosomes with CENH3-depleted centromeres missegregate and partition into micronuclei

Next, we examined the process of HI chromosome loss. Starting with the first (fig. S1B, d) or second (fig. S4C) embryonic mitosis, laggard chromosomes lacking GFP-ts signal appeared in the GEC. Chromatin bridges devoid of GFP-ts signals were detected in early-stage embryos (fig. S5A). Consistent with genome instability reported in other systems (*1*, *11*, *33*–*38*), we also observed micronuclei in early embryos from the GEC (*39*). To determine whether chromosomes in micronuclei carried defective centromeres, we examined the associated centromere states.

Along with 4′,6-diamidino-2-phenylindole (DAPI), the provision of paternal H2B-tdTomato (*14*) provided an unambiguous definition of micronuclei in four–cell stage embryos. In the CC (fig. S5, B and D), all cells carried normal nuclei (micronuclei were absent; *n* = 81 cells from 25 embryos) and most displayed 10 distinct GFP-ts signals. In GEC, 90% (*n* = 29) of embryos harbored up to six micronuclei of varying sizes per embryo, and 59% of them had more than one cell with micronuclei (fig. S5, C and D). Notably, 55% (*n* = 82) of micronuclei had no GFP-ts signals, and the rest carried one to four faint signals. Multiple micronuclei were observed even in later embryo stages (fig. S5, E and F). Thus, the compromised loading of CENH3 and GFP-ts on the centromeres of HI chromosomes led to kinetochore assembly failure, missegregation of laggards, and frequent partitioning within micronuclei similar to those observed in interspecific GECs (*11*, *33*).

### Native CENH3 and GFP–CENP-C display stable inheritance during development of female gametes and zygote

**Fig. 6.**
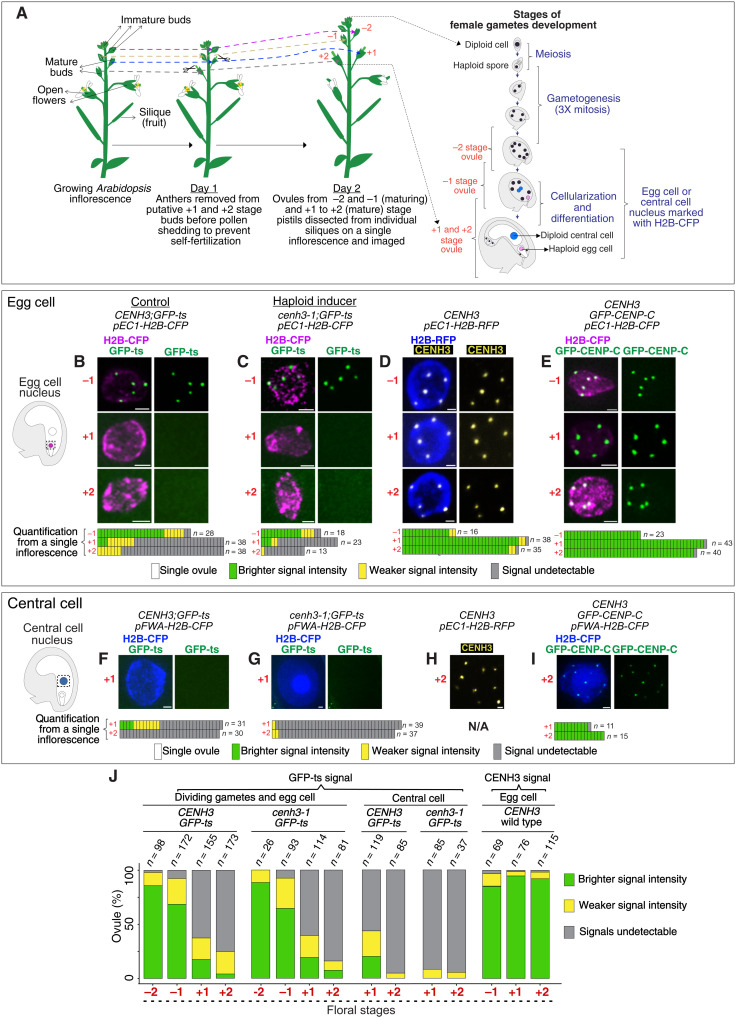
Stability of endogenous CENH3 and CENP-C. (**A**) Experimental setup to study the temporal dynamics of kinetochore proteins during *Arabidopsis* female gamete development. (**B** to **I**) The left drawing indicates the region of interest in the ovule shown on the right. (B to E) Images from a single inflorescence of the respective genotypes from −1 to +2 stage ovules. Quantification of signal type in egg cells or central cells obtained from individual inflorescences is provided below each image (B to G and I). Temporal dynamics of GFP-ts in egg cell nuclei of control (B) and HI (C). (D) CENH3 signals (yellow, by immunostaining) and (E) GFP–CENP-C signals in WT egg nuclei. GFP-ts signals in differentiated central cell nuclei of control (F) and HI (G). (H) CENH3 signals (yellow, by immunostaining) and (I) GFP–CENP-C signals in WT, presumptive central cell nucleus. (**J**) GFP-ts and CENH3 signal quantification in egg and central cells of controls and HI lines. Pooled data for each genotype were collected from at least three independent inflorescences. N/A, not available. Scale bars, 1 μm.

If CENH3 vacates centromeres (*13*), how is centromere identity preserved in the egg and central cell lineage of the CC and not in the GEC? Could GFP-ts be removed selectively while CENH3 persisted? Previous reports are consistent with the possibility of wild-type CENH3 persistence (*12*, *25*, *40*). To compare the stability of GFP-ts and wild-type CENH3, we examined the immature (flower stages −2 and −1) and mature (flower stages +1 and +2) (*41*) ovules bracketing normal self-fertilization time (Fig. 6A; see Materials and Methods). In both control and HI lines, five centromeric GFP-ts signals were visible in developing gametes and immature egg cells but were markedly absent in most mature egg cells (Fig. 6, B, C, and J). However, we detected immunostained WT CENH3 in all egg cell stages (Fig. 6D). Similarly, GFP-ts was absent in mature central cells (Fig. 6, F and G), whereas WT CENH3 was retained (Fig. 6H). Paternally inherited GFP-ts remained visible immediately after fertilization (around 9 HAP; fig. S6B, a) but disappeared thereafter to reload ~25 HAP (fig. S6B, b). However, in the rapidly dividing endosperm, paternal GFP-ts marked all 15 centromeres from fertilization throughout development (fig. S6B, b to d). The removal of GFP-tagged CENH3 from centromeric chromatin is specific to CENH3 since analogous fusions with CENP-C (Fig. 6, E and I, and fig. S6, C to F) and NUF2 (fig. S6G) were retained in the kinetochore through similar stages. In contrast, corroborating observations on wild-type or tagged CENH3 (*13*, *42*), GFP-tagged CENP-C, NDC80, and RFP-tagged NUF2 were removed from the terminally differentiated vegetative nucleus of pollen (fig. S6H).

In summary, in the mature egg and central cell, WT CENH3 persisted in the centromeres while GFP-ts was evicted from both (Fig. 6J). When transmitted by pollen, centromeric GFP-ts was removed in the zygote.

### CENH3 and kinetochore proteins mark one parental set of centromeres in GECs involving other HI CENH3 variants

We asked whether a haploid-inducing, missense *CENH3* mutant (*43*) causes GE through a mechanism similar to GFP-ts. Embryos from *M4* (*CENH3^G83E^*) X WT cross also displayed only five centromeric CENH3 signals in the interphase nucleus (fig. S7A), often along with few faint signals, as seen for *cenh3-1;GFP-ts* X WT. At anaphase, CENH3 localized on the leading chromatids and was absent in the lagging ones (fig. S7B). A missense mutation in another conserved histone fold domain (HFD) residue, CENH3^G173E^, does not act as an HI (*43*). The embryos from this cross displayed 10 bright CENH3 signals and normal segregation (fig. S7, C and D). We then investigated kinetochore assembly in GEC involving these missense mutations and other haploid-inducing CENH3 variants (Fig. 1A). Comparable to GFP-ts (Fig. 5, F and H), embryos from all GECs, including the cross involving the nontransgenic, HI mutant *cenh3-2^A86V^*, displayed bright and faint kinetochore signals (fig. S8, A to F) but with genotype-specific differences. In contrast and corroborating the CENH3 localization pattern (fig. S7, C and D), the non-inducer CENH3^G173E^ variant displayed 10 bright kinetochore signals in a majority of the nuclei (fig. S8G). This suggests that the CENH3^G83E^ variant, like GFP-ts but not CENH3^G173E^, is removed during egg maturation.

M4 (CENH3^G83E^), similar to other CENH3 variants, produces haploids only when maternally transmitted. Correlated with this observation, when M4 and other variants were paternally transmitted, the 5+0 kinetochore pattern was markedly absent except for WT X *cenh3-1;GFP-CENH3*. On the other hand, the 5+5 pattern was more frequent in WT X HI than in the HI X WT GEC (9 to 63% higher; fig. S8, A to E). The increased centromeric signal associated with paternal inheritance suggests a sex bias in CENH3 removal efficiency, also noted with the GFP-ts (Fig. 3, D and E versus G), and provides evidence for function of kinetochores smaller than those of the wild type.

Highlighting a common GE mechanism, native or GE-inducing CENH3s display similar localization bias in the GEC, indicating that only the WT centromeres are competent during the very early embryonic mitoses. If a removal pathway is triggered at egg maturation and persists through the early zygotic stage, then the sex bias for variant CENH3 removal may depend on the different exposure time between egg- and sperm-contributed centromeres.

### Dilution of CENH3 nucleosomes mimics CENH3-dependent HI

**Fig. 7.**
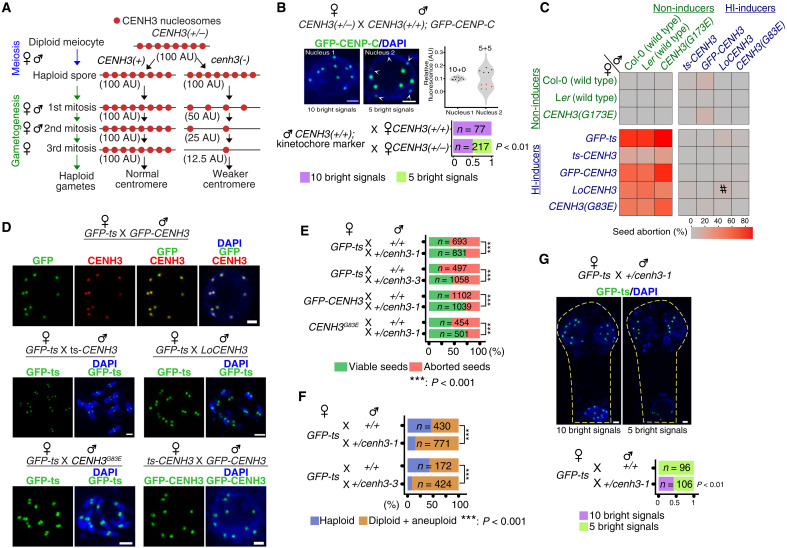
Gametic transmission of a CENH3 null allele mimics the HI CENH3 variants by altering seed death and GE efficiency. (**A**) Hypothetical dilution of centromeric CENH3 during gametogenesis following segregation of WT and null allele in meiosis. (**B**) Patterns and respective counts of kinetochore signal intensity in embryos generated by crossing *CENH3(+/−)* parents with males expressing the kinetochore marker. White arrowheads indicate fainter kinetochore signals. Violin plots display kinetochore signal intensity from the images on the right. Red circles represent fainter signals. Bottom, counts for embryos analyzed in (B). (**C**) Heatmap of seed abortion (a proxy for GE frequency) when intercrossing non-inducer and HI lines. #Data from the work of Maheshwari *et al.* (*7*). *GFP-ts* was not used as male because of reduced fertility. (**D**) Normal, 10-chromosome loading of GFP-ts or GFP-CENH3 crosses between *GFP-ts* and *GFP-CENH3* with other HI lines (interphase or prometaphase stage nuclei). (**E**) Comparison of proportion of viable and aborted seeds upon pollinating various HI lines with *CENH3(+/+)* versus *CENH3(+/−)* males. (**F**) Bar graph displays GE induction efficiency when pollinating *cenh3-1;GFP-ts* with *CENH3(+/−)* males. (**G**) Embryos from *cenh3-1;GFP-ts* X *CENH3(+/−)* with two patterns of GFP-ts signal. Counts are displayed in the bar graph at the bottom. Scale bars, 1 μm. Statistical validation was by the Chi square test.

**Fig. 8.**
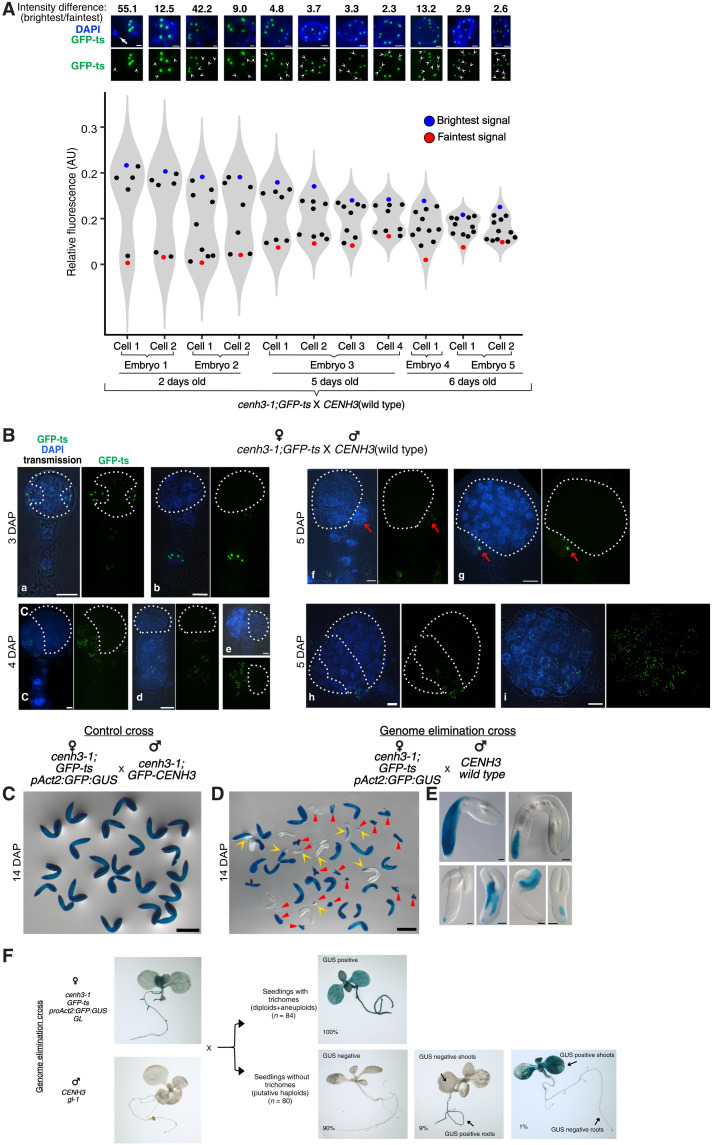
Centromeric resilience and stochastic nature of chromosome elimination in GEC. (**A**) Resiliency of HI centromeres demonstrated by progressive coalescence of bright and faint GFP-ts fluorescence intensity clusters. Note the decrease in the maximum-minimum difference between HI centromeres and WT centromeres in different embryos from 2 to 6 DAP of the GEC (white arrowhead, weaker signals; white arrow, micronuclei). (**B**) The GFP-ts protein is encoded by the HI genome and can be used to document GE. In the GEC [(B), a to h], early embryos display chimeric distribution of GFP-ts signals, indicating GE in cells lacking any signal or displaying fainter signals (highlighted with dotted white line). Compare this to an embryo that avoided GE and displays GFP-ts signals in all nuclei [(B), i]. Red arrow: A GFP-ts–positive cell at the base of embryo proper in an otherwise GFP-ts–negative embryo, suggestive of missegregation of HI chromosomes during early stages of embryo development. (**C** to **F**) GUS histochemical staining analysis. GUS signal is seen uniformly in 2-week-old control embryos (C). Note that these embryos are from the *cenh3-1;GFP-ts* X *cenh3-1;GFP-CENH3* cross, which does not result in GE (see Fig. 7C). (D) GUS staining chimerism (yellow arrowheads) indicates incomplete GE during embryo development. Smaller embryos (red triangles on a subset) likely result from severe aneuploidy or defective endosperm. (E) Chimeric embryos at higher magnification. (F) Whole-seedling GUS staining revealing the chimeric nature of GE. Seedlings with trichomes constitute either the hybrid diploid or aneuploid progeny, while those without trichomes are predominantly haploid. *n*, number of seedlings. Scale bars, 1 μm (A), 5 μm (B), 500 μm (C and D), and 50 μm (E).

Depletion of altered CENH3 may dilute the density of CENH3 nucleosomes below a threshold, diminishing the competitiveness of the affected centromeres (*44*). Furthermore, biased loading in 1 of 11 embryos from the interploidy cross 4x *CENH3(+/−);GFP-ts* X 2x WT suggested a centromeric defect in gametes inheriting the *cenh3-1* null allele. Accordingly, we tested the possibility of CENH3 depletion using diploid plants heterozygous for the *cenh3-1* knockout mutation (*CENH3/cenh3-1*). Following meiosis, the female and male haploid spores undergo three and two mitoses, respectively, to produce gametes. Spores inheriting the null allele should progressively deplete CENH3 (Fig. 7A). In contrast to maize (*3*), the *cenh3-1* null allele is normally transmitted in *Arabidopsis* (*45*). However, when a *CENH3/cenh3-1 Arabidopsis* was crossed to the *CENH3(+/+)* as male or female, half of the two– to four–cell stage embryos displayed biased kinetochore loading (5+*N*; *N* = 1 to 5), while the remaining half displayed 10+0 pattern (Fig. 7B and fig. S9A). The 5 bright + 5 faint signal pattern was retained even in >16–cell stage embryos [3 days after pollination (DAP); fig. S9B]. In contrast, all embryos from *CENH3(+/+)* parents displayed the 10+0 pattern (Fig. 8B and fig. S9A). On the basis of these observations, we concluded that the centromeres carrying the faint signals in embryos with the 5+*N* pattern originated from gametes carrying the *cenh3-1* null allele. Progeny from *CENH3/cenh3-1* X *CENH3(+/+)* yielded 4 haploids/956 progeny or 0.83% of zygotes formed by (−) eggs. None were found in control (*n* = 1207 progeny). This highlights the importance of threshold CENH3 concentration in centromere function and demonstrates that haploids can be induced without altering CENH3 but by merely diluting its WT form. Production of haploids from maize heterozygous for CENH3 was demonstrated by Wang *et al.* (*3*) who proposed that dilution was responsible.

### Gametic transmission of a CENH3 null allele mimics the HI CENH3 variants by altering seed death and GE efficiency

We searched for factors that affect CENH3-mediated HI using seed death to quantify GE efficiency (*2*, *6*, *22*). Expanding on our previous observations (*22*), the GFP-ts HI’s best suppressors were found to be another CENH3-based HI, including fusion proteins, point mutations, and diverged CENH3. The majority of the HI X HI cross generated only a background level (0.3 to 3%) of seed death (Fig. 7C), a trait strongly associated with HI efficiency (*6*). Corroborating these observations, most embryos from *GFP-ts* or *GFP-CENH3* X other HIs displayed a uniform 10+0 pattern (Fig. 7D), a notable deviation from the *GFP-ts* X WT cross (Fig. 3, D and E). Thus, the 10+0 pattern provided a visual assay for functional recovery of centromeres inherited from GFP-ts and other HIs. Consistent with these observations, the CENH3-depleted *cenh3-1* male gametes from *CENH3/cenh3-1* plants were good suppressors of seed death, reducing it by 30% in three different HIs (Fig. 7E). Similar results were observed in an independent null allele, *cenh3-3* (Fig. 7E). Correlating with reduced seed death, fertilization by gametes inheriting the *cenh3-1* null allele from *CENH3/cenh3-1* or *CENH3/cenh3-3* reduced the haploid frequency by ~30% (Fig. 7F), presumably by matching the epigenetic strength of the female’s GFP-ts–depleted centromeres (Fig. 7C). This is consistent with the appearence of 10+0 pattern in 42% of the early-stage embryos (Fig. 7G), a feature characteristic of the CC and never observed in the GEC.

### Alternative fates of chromosomes with CENH3-depleted centromeres

Centromeres can replenish centromeric-CENH3 after initial depletion (*44*, *46*, *47*). The underperformance of HI chromosomes in early embryos, the associated fainter signals, and the production of diploid and aneuploid progeny from a GEC suggested the recovery of centromere function in HI chromosomes during embryo development. Supporting our hypothesis, comparing nuclei from independent embryos in the 2 to 6 DAP window, we observed progressive convergence of high and low GFP-ts signals toward uniformity (Fig. 8A). In addition, HI chromosomes carrying fainter GFP-ts signals appeared to be segregating normally (fig. S5, A and F). At the same time, GE appeared stochastic, as highlighted by frequent chimerism in GFP-ts signals carrying nuclei (Fig. 8B). We used the histological marker β-Glucuronidase (GUS) provided as a transgene in the HI genome to visualize the GE pattern in later-stage embryos. At 14 DAP, embryos in a non-GEC (HI X HI) displayed uniform development and staining (Fig. 8C), while GEC’s embryos varied widely in development and staining pattern (Fig. 8, D and E). Later, chimerism was also common in seedlings, as revealed by its variable size and discontinuous staining (Fig. 8F). We concluded that shoot apical meristem state predicts progeny’s ploidy upon germination. Together, if HI chromosomes escape early missegregation, then their centromeres become progressively more competent for CENH3 loading.

### Null mutants of VIM1 enhanced haploid induction frequency

**Fig. 9.**
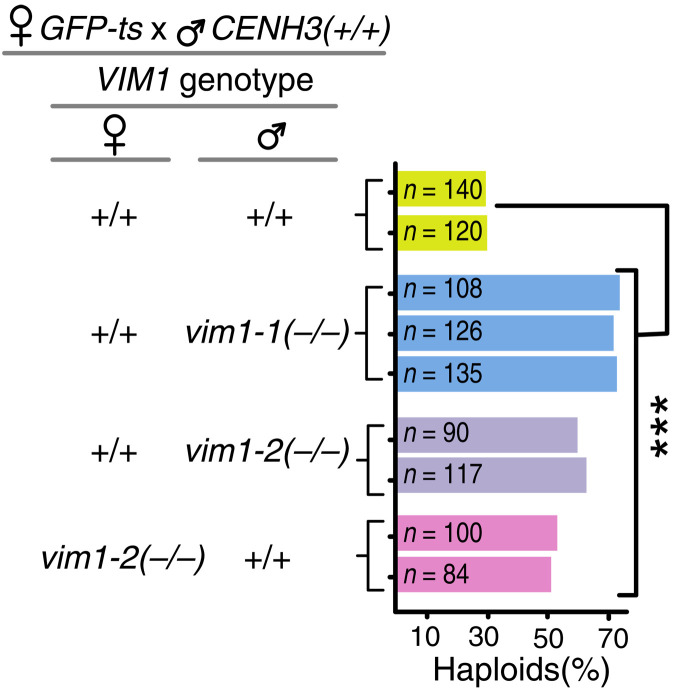
Null mutants of VIM1 enhance haploid induction frequency. Bar graph showing the effect on haploid induction of null alleles of *VIM1* transmitted from either parent. “*n*” indicates the number of progeny scored. P of Chi square test < 0.001

To identify factors affecting GE frequency, we undertook a genetic screen exploiting natural variation in *Arabidopsis* germplasm. Among the 20 diverse accessions crossed as a male parent in the GEC, accession Bor-4 produced higher haploid induction frequency (~70%) compared to ~30% with the CC involving accession Col-0 (Fig. 9). The Bor-4 accession carries a deletion of *VIM1* (*vim1-1*), a methyl cytosine binding protein that results in hypomethylation of centromeric repeats, centromeric decondensation, and decreased CENH3 density at the centromeres (*48*–*50*). VIM1 displays homology to the yeast E3 ubiquitin ligase PSH1 (*49*, *51*, *52*), which regulates stability of yeast centromeric histone CSE4. To further rule out background genetic or epigenetic effects resulting from the natural accession Bor-4, we tested whether the null allele *vim1-2* in the Col-0 background can also have a similar effect on GE. We found that the *vim1-2* allele also acted as an effective enhancer of haploid induction when transmitted by either parent (Fig. 9), suggesting that a critically low level of VIM1 in the zygote engenders centromeric failure. The strong effect of these modifiers indicates that the ubiquitination or methylation pathway, directly or indirectly, affects CENH3 stability.

## DISCUSSION

**Fig. 10.**
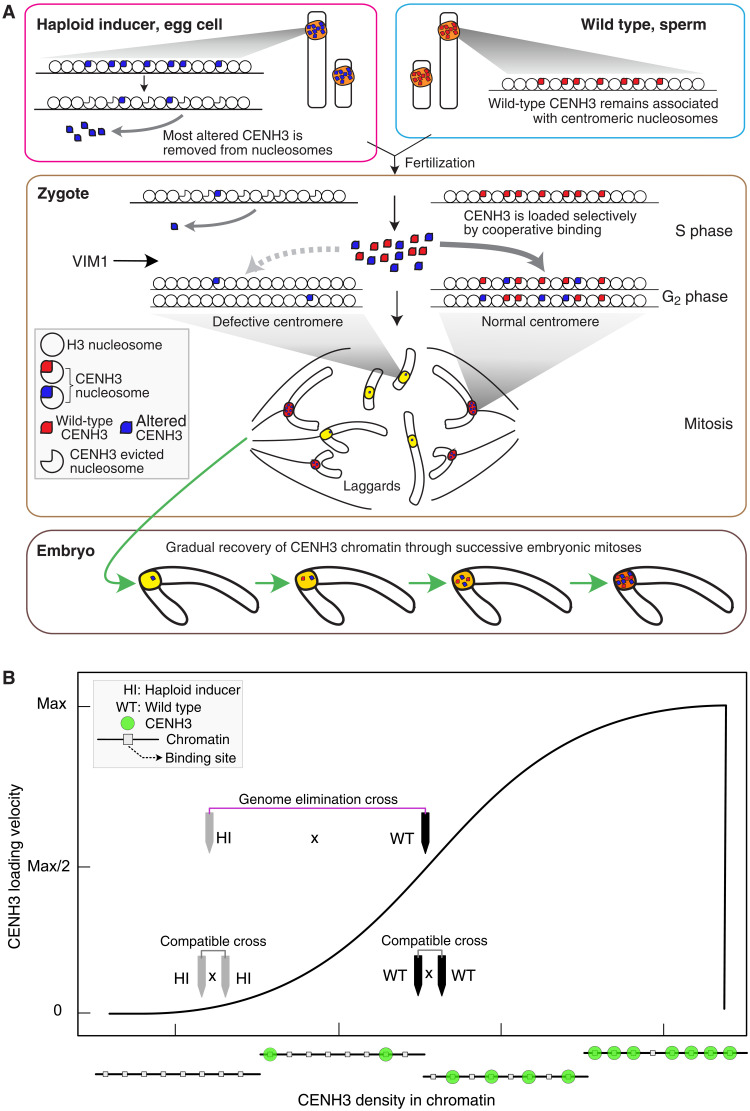
Model for CENH3-mediated haploid induction in *Arabidopsis*. (**A**) Altered CENH3 is removed selectively from chromatin at the mature egg stage. WT CENH3 is not. In the cell divisions following hybridization, CENH3-depleted “weak” centromeres must compete for CENH3 loading with WT centromeres. Because of cooperative binding, WT centromeres are favored and load CENH3 preferentially. In the ensuing mitosis, the HI chromosomes missegregate because of their weak centromeres. The recessive action of altered CENH3 is explained by the persistence of WT CENH3. The action of VIM1 favors CENH3 loading by an unknown mechanism. We further hypothesize that selective removal results from a genomic surveillance mechanism that eliminates defective or misplaced CENH3 molecules. (**B**) Cooperative binding of CENH3 to centromeres according to Hill-Langmuir kinetics. Plot of CENH3 loading velocity on chromatin as a function of CENH3 density in chromatin. Binding sites represent the possible normal location of CENH3 nucleosomes in a regular, CENH3-rich centromere, which are interspersed between regular nucleosomes (*54*, *74*). Velocity = 0 when all sites are either empty or occupied. According to the model, at the onset of GE, parental centromeres differ in density of CENH3 (compare gray and black wedges to chromatin reference drawings at the bottom) and are loaded differentially (plotted response graph). When both parents contribute similarly depleted centromeres (gray wedges), loading is initially slower but proceeds at the same rate on both parental centromeres, ensuring balanced loading and a compatible outcome in the cross.

Our results suggest a model (Fig. 10A) where a surveillance system (*51*–*53*) recognizes and removes CENH3 variants that are mismatched with coevolved centromeric factors. In addition to removal from egg, central cells, and zygote, our results and those of Ingouff *et al.* (*13*) indicate that removal and reloading of GFP-ts also takes place in male meiocytes (*40*). The CENH3-depleted centromeres maintain some centromeric chromatin identity, probably because removal of CENH3 is incomplete or an associated chromatin mark remains (*31*) and may guide CENH3 loading. When both parents contribute CENH3-depleted centromeres (HI self-cross or HIxHI cross), the absence of competition between centromeres enables the CENH3 chromatin to regain uniform and optimal CENH3 levels. In contrast, in the HI X WT cross, depletion of CENH3 variants from the HI centromeres results in a large epigenetic imbalance with WT centromeres, which maintain the CENH3 mark. During zygotic loading of CENH3, the CENH3-depleted chromosomes of the HI compete poorly with WT ones, as suggested after CID depletion in fruit fly (*44*). We hypothesize that cooperative binding kinetics favor centromeres with a high density of CENH3 nucleosomes (Fig. 10B). A similar problem, the preferential incorporation of CENP-A in CENP-A–rich chromatin, has been explained by mass action kinetics (*54*). Because of the constraints of mass action kinetics in modeling the differential binding of an equal concentration subunit (CENH3) to a nonsoluble, stable complex (the centromere) with different density of bound subunits (*55*), the model proposed to explain the cooperative binding of Polycomb complex subunits or the even-skipped repressor to multiple binding sites in DNA (*56*, *57*) better fits our observations and model. This epigenetic imbalance can be established when selfing heterozygotes for a null CENH3 allele, explaining the function of maize and wheat HIs (*3*, *4*) and, potentially, GE in barley (*11*).

HI centromeres retain a weak but distinct epigenetic memory. Many zygotes in GECs eventually form diploids or aneuploids, demonstrating resilience of the depleted centromeres and providing a model for centromere recovery. Ploidy chimerism at all embryo developmental stages suggests stochastic GE explaining formation of haploid, diploid, and aneuploid plant types (*39*). Because different CENH3 variants form an allelic series varying in GE efficiency (*6*, *7*), the epigenetic strength of centromeric identity and the potential for recovery must vary proportionally with the removal efficiency of each variant in the egg cell.

In crosses that do not lead to measurable HI, such as WT X *GFP-CENH3* (*2*) and WT X *M4 (CENH3^G83E^)* (*6*), in two- to four-cell embryos, we observed five normal centromere signals, presumably contributed by the WT, and five small centromere signals, presumably contributed by the potential HI. Contrasted to GEC, where the HI centromeres are mostly undetectable, this indicates that *Arabidopsis* embryos can tolerate large differences in parental kinetochore size and only severe CENH3 depletion on one parental centromere set results in efficient HI. Therefore, in *Arabidopsis*, reduction of one parent’s kinetochores to a very small but detectable size does not trigger HI as proposed (*58*).

Mutations of the epigenetic factor VIM1, which can ubiquitinate CENH3 in vitro and affects both DNA methylation and chromocenter size (*48*, *49*), markedly increased GE efficiency. The effect manifested when either parent was the *vim1* mutant, suggesting that a critical level of VIM1 postzygotic activity is needed to stabilize CENH3 or facilitate loading, either through a stabilizing ubiquitin mark or through differential DNA methylation. This VIM1 effect appears opposite to what would be expected from the action of its homolog PSH1, which entails removal of ectopic and unchaperoned CSE4 (the yeast centromeric histone homologous to CENH3) via ubiquitination (*52*). Whether VIM1 confers stability to CENH3 via ubiquitination as reported for CENP-A (*59*) or acts indirectly remains to be determined. Directly or not, a ubiquitin pathway rescues centromere failure.

The property of CENH3 mutations described here could have interesting evolutionary implications. There is good evidence for the requirement of an optimal, species-specific CENH3 structure (*60*). In *Arabidopsis*, evolutionary divergence of the complementing CENH3 results in increasing GE efficiency (*7*), suggesting a progressively more severe mismatch with coadapted factors (*61*–*63*). Changes in CENH3 structure may expose CENH3 to a surveillance system whose presence is well established in yeast and humans (*51*–*53*, *64*). In this context, species differences are likely. The efficient centromeric function in crosses between CENH3-dependent HIs suggests that evolution of species with subefficient CENH3 function is possible. For example, the *CENH3^G83E^* mutation resulting in GE could persist at low frequencies because it is recessive. Rarely, it may become fixed in a geographically isolated subpopulation without affecting short-term fitness, as suggested by surveys (*43*). Lethality in the HI X WT cross should reinforce speciation by establishing a strong postzygotic barrier while, at the same time, fostering novel karyotypes (*43*). In the wild type, CENH3 density–dependent competition may help maintain dominance of the centromere over potential ectopic loci seeded by CENH3. The presence of a CENH3 variant, however, could increase the threat by neocentromeres if the reduced difference between the centromere and secondary CENH3 loci lessens the bias in loading. Selection against the resulting genome instability would favor compensatory changes in CENH3 and interacting kinetochore proteins and perhaps help explain the rapid evolution of CENH3 (*60*).

The difficulty in replicating the *Arabidopsis* HI system in crops (*3*, *4*) could be explained by species-specific variation in quantitative or developmental features of mechanisms that regulate CENH3 deposition and stability. The GFP-ts alteration is tolerated in *Arabidopsis* but lethal in maize (*3*). At the same time, transmission of a null *cenh3* allele yields 5% haploids in maize, <1% in *Arabidopsis*, and none in wheat (*3*, *4*). Designing an efficient HI in each species may require different CENH3 modifications, as different constraints could apply to the function of CENH3 variants. Direct manipulation of CENH3 removal in the gametes may constitute a more general strategy (*44*).

In conclusion, our analysis of CENH3 variant–mediated haploid induction in *Arabidopsis thaliana* demonstrates its dependency on selective destabilization of HI-CENH3 variants during fertilization. The resulting differences in CENH3 stability provide insight into mechanisms that maintain the epigenetic memory of centromeric chromatin. Together, these findings provide a firm basis for further mechanistic insights and a framework for evaluating mechanisms of GE in biotechnology and during distant hybridization.

## MATERIALS AND METHODS

### Plant growth and materials

*Arabidopsis* plants were grown in standard long day photoperiod (16-hour light/8-hour dark) conditions at 20°C in the controlled environment facility at University of California, Davis (UC Davis). Unless mentioned otherwise, all lines used in the study were homozygous for the genotype of interest. The lines carrying the *cenh3-1* and *cenh3-3* null allele were maintained and used in heterozygous condition because of the embryo-lethal nature of the null mutations. The following lines used in this study have been previously described: *CENH3/cenh3-1*, *GFP-ts*, *GFP-ts;CENH3*, *GFP-CENH3*, *tailswap-CENH3* (the Chan Laboratory) (*2*, *45*); *LoCENH3* and *ZmCENH3* (Comai Laboratory) (*7*, *24*); *CENH3^G83E^*(M4) and *CENH3^G173E^*(M47), *cenh3-2*(*CENH3^A86V^*) (Britt laboratory) (*43*); and *vim1-1* (Bor-4, CS22591) and *vim1-2* (SALK 000930c) (*48*) from the Arabidopsis Biological Resource Center (ABRC). Zygosity of the transgenes in *tailswap-CENH3* (Simon laboratory) (*2*, *45*), *LoCENH3* and *ZmCENH3* (Comai Laboratory) (*7*, *24*), *CENH3^G83E^*(M4), and *CENH3^G173E^*(M47) lines are unknown. Lines carrying *proEC1-H2B-RFP* and *pro HTR10-HTR10-RFP* (RFP-tagged histone H3.3 variant) (*13*) were provided by F. Berger (Gregor Mendel Institute, Austria). The line carrying *pRPS5A-H2B-tdTomato* (*14*) was provided by T. Higashiyama (Nagoya University). A population of *4xGFP-ts* segregating for *cenh3-1* and *CENH3* alleles was provided by N. De Storme, Geelen laboratory (University of Ghent). Col-0 (2x), L*er gl-1*(2x) (S. Chan Laboratory stocks), and L*er* 4x (Comai Laboratory stock) were used as WT strains in the context of *CENH3*. *CENH3;mCherry-ts*, *GFP-CENP-C*, *NUF2-GFP*, and *NDC80-GFP* are unpublished lines from the Chan Laboratory. Natural accessions (see below) used in screening for modifiers of HI frequency were obtained from ABRC. The following lines used in this study were generated by the floral dip method for *Arabidopsis* (*65*) using corresponding constructs generated with *pCAMBIA1300* or *pCAMBIA3300* vector backbone in the Chan Laboratory and the Comai Laboratory: *proFWA-H2B-eCFP*, *proEC1-H2B-eCFP*, *proCENP-C-TagRFP-CENP-C* (referred as *RFP-CENP-C* in the text), *proNUF2-NUF2-TagRFP* (referred as *NUF2-RFP* in the text), *proH2B-H2B-CFP* (At5g22880), and *proAct2-NLS-GFP-GUS*. The *cenh3-3* [23–base pair (bp) deletion from +1177 to +1199 bp] null mutant was generated in L*er*(*gl1*) ecotype using *pKAMA-ITACHI Red CRISPR-Cas9* system (*66*) with single guide (5′-CCCCTCCCCAAATCAATCGT-3′) targeting the eighth exon. The transgenic locus (Cas-9 and guide RNA) were segregated out in the T_2_ generation. T_3_ generation lines carrying the *cenh3-3* allele were used for the experiments in this study. Additional details of all plasmid constructs and strains used in this study will be provided upon request.

### Emasculation and pollination

For crossing and imaging, the mature flower buds were identified and emasculated a day before anther dehiscence. The following day, either the ovules from the pistils of the emasculated buds were directly imaged (see below) or pollinated with appropriate male genotypes for imaging double-fertilization events and for examining various stages of embryo and endosperm development as described below.

### Ovule and embryo dissection

At selected time points, ovules from individual pistils were dissected using insulin needles directly into a drop of mounting media [1× phosphate-buffered saline (PBS) and 50% glycerol] on the glass slides; a coverslip was gently placed on top of it, and corners were locked in position with nail polish. The volume of mounting media was found critical for proper imaging. We typically use 15- to 25-μl volume per 22 mm × 22 mm cover glass, and the volume depends on the quantity and size (age) of the ovule. Higher volume increases the thickness of the tissue (in the *z* axis), which results in poor signal quality. In contrast, below a threshold volume, the ovules may get smashed and gametic, or endosperm nuclei may be disfigured or released through the micropyle. For a subset of immunostaining experiments, before embryo isolation, inflorescence with intact crossed pistils were soaked in solution with 2 mM 8-hydroxyquinoline and 0.25% of colchicine for 2 to 3 hours to enrich cells with metaphase stages. Embryos were manually dissected from the fertilized ovules from 2 to 14 DAP under a stereo microscope using a fine tungsten needle while immersed in 0.1× PBS solution. While dissecting four–cell stage embryos (two cells in the embryo proper and two cells in suspensor), often, the bottom cell or both cells in the suspensor get severed. The dissected embryos were transferred to glass slides with ~5 μl of the same buffer using a fine glass tube or 10-μl plastic pipette tip coated with bovine serum albumin (100 mg/ml). Leaving 2 to 3 μl of buffer with embryos, the rest of the medium was removed and the mounting medium [3 to 5 μl of 1× PBS and 50% glycerol with DAPI (1 μg/ml)] was quickly added to avoid drying of the tissues. A coverslip was gently placed on top of the samples for direct observation. Mounted embryo or ovule samples were imaged on the same day of preparation. For immunostaining, dissected embryos or ovules were transferred to a glass slide in a drop of 0.1× PBS and processed further as described below.

### Staging flower buds for imaging and immunostaining

For imaging the chronological dynamics of GFP-ts, WT-CENH3, and CENP-C-GFP during female gamete development, unopened buds from an inflorescence axis were assigned negative(− sign) numbers and open flowers were given positive(+ sign) numbers (Fig. 6). In our growth conditions, every inflorescence axis produces more than one flower per day. Hence, we used numerical nomenclature with minus and plus signs (*41*) to specify relative stages of the buds and flowers instead of standard staging nomenclature for *Arabidopsis* based on flower (*67*) or ovule development (*68*). On a given inflorescence axis, the −1 stage being the matured bud and −2 stage is chronologically younger and immediate in the order of the acropetal succession. In contrast, the +1 stage refers to chronologically young but open flower, and +2 stage refers to chronologically immediate and older open flower in basipetal order (Fig. 6A). The buds in the −2 stage contain both undifferentiated gametes and differentiated egg cells. Whereas, −1 stage buds contain predominantly differentiated egg cells. The +1 and +2 stage flowers predominantly carry ovules with differentiated egg and central cells that are ready for fertilization.

### Immunostaining of nucleus and embryos

Nuclei from two to three formaldehyde-fixed young flower buds were extracted in PBS by fine chopping using a sharp razor blade. Chopped tissue was resuspended in 1 ml of cold PBS and filtered through a 40-μm nylon strainer. Nuclei in the filtrate were concentrated by centrifugation (250*g* for 5 min). Leaving ~15 μl of the supernatant along with the nuclei pellet, the rest of the supernatant was gently removed and discarded. The nuclei pellet was gently resuspended by pipetting up and down using a wide-bore pipette tip. One to two microliters of nuclei suspension was used for immunostaining. Immunostaining on isolated nucleus, dissected embryos, and whole-mount ovules was performed according to (*69*) with minor modifications. Immunostained samples were mounted with ProLong Gold antifade with DAPI (Thermo Fisher Scientific) before imaging. Antibodies used in immunostaining are as follows: primary, rabbit CENH3 (1:2000) (*23*), GFP (1:400; #ab6556), H3K4Me3 (1:500; #07-473-Milipore), H3K9me2 (1:200; #ab1220), and H3S10ph (1:100; #ab14955) and secondary, Alexa Fluor 405, 488, 594, and 647 from Invitrogen (used in 1:100 to 1:500 dilutions).

### GUS staining

GUS staining of embryo and seedling was carried out as described (*70*).

### Visualization of cells from different reproductive stages using cell-specific chromatin markers

For imaging postfertilization ovules, the egg cell chromatin was labeled with histone H2B-CFP (cyan fluorescent protein; At5g22880) or H2B-RFP (*13*) and the central cell or endosperm is marked by H2B-CFP (At5g22880) fusion proteins. For labeling the egg cell, zygote, and two-cell embryo, the fusion proteins H2B-CFP or H2B-RFP were driven by *EC1*, an egg cell–specific promoter (*19*), and for labeling the central cell and endosperm nuclei, the fusion protein was driven by *FWA*, a central cell and endosperm-specific promoter (Figs. 1 and 6 and figs. S1, S2, and S6) (*71*). The sperm chromatin was marked by H2B-tdTomato or HTR10-RFP (histone H3 variant) fusions driven by *RPS5A* promoter (*14*) or sperm cell–specific *HTR10* promoter, respectively (Fig. 1 and figs. S1, S2, and S6) (*13*). Following fertilization, in *pEC1-H2B-CFP* X *pHTR10-HTR10-RFP* crosses, the zygotes were identified by colocalization of H2B-CFP and HTR10-RFP signals whereas endosperm were exclusively marked by HTR10-RFP from the male. Similarly, in *pFWA-H2B-CFP* X *pHTR10-HTR10-RFP* crosses, endosperm was identified by colocalization of H2B-CFP and HTR10-RFP signals whereas zygotes were marked exclusively by HTR10-RFP. Similar method was used to identify the endosperm and embryo while using *pRPS5A-H2B-tdTomato*–expressing males. For imaging pollen, pollen grains from the open flower were directly dusted onto mounting media [1× PBS and 50% glycerol with DAPI (1 μg/ml)], and a coverslip was gently placed on top and imaged directly. Sperm nuclei were differentiated from the vegetative nuclei by bright and smaller DAPI signal in contrast to the relatively larger diffused signal from the latter.

### Microscopy and image analysis

Fluorescence images of nucleus, ovule, embryo, and pollen samples were captured as 3D objects using Applied Precision DeltaVision deconvolution or spinning disk confocal microscope in MCB light microscope imaging facility (UC Davis). With the Applied Precision DeltaVision deconvolution microscope, images were captured at 60× magnification with *Z*-stacks (with a step size of 0.2 μm for embryos and up to 1 μm for ovules for initial screening and 0.2 to 0.5 μm on selected images). One selected prometaphase sample (Fig. 4C) was imaged with Nikon Structured Illumination Microscope for higher resolution. Zeiss Discovery v20 stereoscope was used to image seeds, seedlings, and GUS-stained samples.

Images captured with Applied Precision DeltaVision deconvolution microscope were deconvolved with DeltaVision softWoRx. All 3D images were analyzed with Imaris (for images acquired with Applied Precision DeltaVision) and SlideBook (only for 3i SDC image acquisition) software. During analysis, if needed, a series of consecutive *z* planes were analyzed for resolving overlapping signals. On selected 3D images, only *z*-stacks with cells or tissue of interest were selected and transformed into 2D using maximum intensity projection (MIP) method. Those selected images were processed using Adobe Photoshop, and figures were assembled with Affinity Designer. Images captured using Zeiss Discovery v20 stereoscope were processed using ZEN lite software (Zeiss). While processing and analyzing images, brightness and contrast were altered and uniformly applied to the whole images to (i) reduce the background noise, (ii) make the fainter fluorescence signals visible relative to the brighter signals in the same image, and (iii) to reduce the background autofluorescence in the ovule whole mounts.

To prepare all figures in the manuscript, we selected representative images from each experiment, but wherever possible, we chose images that had no overlapping centromere or kinetochore signals upon transforming to 2D format (MIP). The same criteria were used to select the images for signal intensity analysis presented in Figs. 2, 3, 5, 7, and 8 and figs. S3 and S9. Centromeric signal (fluorescence) intensity of GFP-ts, GFP-CENH3, CENH3 (by immunostaining), RFP–CENP-C, and NUF2-RFP was measured using SoftWoRx Explorer (Applied Precision Inc.) on selected raw images with non-overlapping signals in the region of interest. By scanning through the *z* axis for each 3D image, a 5 × 5 pixel area was selected with the region of interest, aligned at the center, and the intensity maximum from the point spread function for each signal was recorded (*72*). Background noise was removed by selecting the same size region next to the region of interest for each signal. Given that the analyzed cells may be in unknown stages of the cell cycle (except G_2_-anaphase), collected centromeric fluorescence values [arbitrary units (AU)] were normalized within each analyzed cell and expressed as relative fluorescent intensity with AU. Qualitative patterns of centromeric GFP or RFP signal intensity were assigned by visual inspection of individual nuclei or embryos of interest using Imaris software. Graphs were generated with RStudio. The egg and central cells were readily identified by the expression of the H2B-CFP marker driven by EC1 and FWA promoters, respectively, in addition to their developmental position as gauged by the virtue of autofluorescence of ovule integuments (*13*, *18*). The centromeric GFP-ts signals in the undifferentiated gametes were recognized by the presence of GFP signals in a group of five (gametic chromosome number) in the mid-sections toward the micropylar end of the ovule, which is otherwise free of somatic cells. The CENH3 immuno signals in presumptive central cells in the WT background are recognized by its proximity to the egg cell and in the mid-sections of the ovule (Fig. 6H).

### Seed death and haploid induction

In the GECs, seed death can be used as an indirect measure of haploid induction efficiency (*6*). For most of the experiments, crossed seeds were collected from individual silique (fruit) and totaled following the quantification. For the seed death shown in Fig. 7C for all of the cross combinations [except WT (Col-0) X *GFP-ts* (*n* = 87) and *GFP-CENH3* X *M47(CENH3^G173E^)* (*n* = 48)], a minimum of 240 and a maximum of 923 seeds were examined. The haploid frequency was scored by phenotyping the progeny as described in (*2*, *43*).

### Screen for modifiers of HI frequency

A total number of 20 geographically diverse natural accessions of *A. thaliana* were used in the modifier screen: Sha-1(CS6180), Fei-0(CS22645), Cvi-0(CS1096), Hi-0(CS1226), KNO-1(CS22401), Pro-0(CS22649), Rsch-0(CS28715), Lago-I(CS76367), Rmx-A02(CS22568), Col-0, L*er*, Angel-1(CS76362), Sij-2(CS76380), Ey15-2(CS76399), IP-Bus-0(CS76736), Cnt-1(CS1635), Uod-1(CS22612), Altai-5(CS76433), RRS-7(CS22564), and Bor-4(CS22591). All these accessions were obtained from ABRC, Ohio State University and were shared with us for the screen by J. Kaur (University of Delhi, South Campus, India) and D. Barua (Indian Institute of Science Education and Research, Pune, India). All the listed accessions were crossed as a pollen parent to *cenh3-1;GFP-ts* female, and the seeds from the resultant crosses were harvested upon maturation. The viable seeds were then germinated on MS-agar growth medium and subsequently transplanted to the soil for growth. The percentage of haploid, diploid, and aneuploid progeny was scored as previously described (*2*, *43*, *73*).

## Notes

View/request a protocol for this paper from *Bio-protocol*.

## References

[1] T. Ishii, R. Karimi-Ashtiyani, A. Houben, Haploidization via chromosome elimination: Means and mechanisms. Annu. Rev. Plant Biol. 67, 421–438 (2016). 26772657 10.1146/annurev-arplant-043014-114714

[2] M. Ravi, S. W. L. Chan, Haploid plants produced by centromere-mediated genome elimination. Nature 464, 615–618 (2010). 20336146 10.1038/nature08842

[3] N. Wang, J. I. Gent, R. Kelly Dawe, Haploid induction by a maize *cenh3* null mutant. Sci. Adv. 7, eabe2299 (2021). 33523932 10.1126/sciadv.abe2299 PMC7817090

[4] J. Lv, K. Yu, J. Wei, H. Gui, C. Liu, D. Liang, Y. Wang, H. Zhou, R. Carlin, R. Rich, T. Lu, Q. Que, W. C. Wang, X. Zhang, T. Kelliher, Generation of paternal haploids in wheat by genome editing of the centromeric histone CENH3. Nat. Biotechnol. 38, 1397–1401 (2020). 33169035 10.1038/s41587-020-0728-4

[5] T. Fukagawa, W. C. Earnshaw, The centromere: Chromatin foundation for the kinetochore machinery. Dev. Cell 30, 496–508 (2014). 25203206 10.1016/j.devcel.2014.08.016 PMC4160344

[6] S. Kuppu, M. Ron, M. P. A. Marimuthu, G. Li, A. Huddleson, M. H. Siddeek, J. Terry, R. Buchner, N. Shabek, L. Comai, A. B. Britt, A variety of changes, including CRISPR/Cas9-mediated deletions, in CENH3 lead to haploid induction on outcrossing. Plant Biotechnol. J. 18, 2068–2080 (2020). 32096293 10.1111/pbi.13365 PMC7540420

[7] S. Maheshwari, E. H. Tan, A. West, F. C. H. Franklin, L. Comai, S. W. L. Chan, Naturally occurring differences in CENH3 affect chromosome segregation in zygotic mitosis of hybrids. PLOS Genet. 11, e1004970 (2015). 25622028 10.1371/journal.pgen.1004970 PMC4314295

[8] R. Karimi-Ashtiyani, T. Ishii, M. Niessen, N. Stein, S. Heckmann, M. Gurushidze, A. M. Banaei-Moghaddam, J. Fuchs, V. Schubert, K. Koch, O. Weiss, D. Demidov, K. Schmidt, J. Kumlehn, A. Houben, Point mutation impairs centromeric CENH3 loading and induces haploid plants. Proc. Natl. Acad. Sci. U.S.A. 112, 11211–11216 (2015). 26294252 10.1073/pnas.1504333112 PMC4568683

[9] N. C. Subrahmanyam, K. J. Kasha, Selective chromosomal elimination during haploid formation in barley following interspecific hybridization. Chromosoma 42, 111–125 (1973).

[10] K. Mochida, H. Tsujimoto, T. Sasakuma, Confocal analysis of chromosome behavior in wheat x maize zygotes. Genome 47, 199–205 (2004). 15060616 10.1139/g03-123

[11] M. Sanei, R. Pickering, K. Kumke, S. Nasuda, A. Houben, Loss of centromeric histone H3 (CENH3) from centromeres precedes uniparental chromosome elimination in interspecific barley hybrids. Proc. Natl. Acad. Sci. 108, E498–E505 (2011). 21746892 10.1073/pnas.1103190108 PMC3158150

[12] T. Ishii, N. Sunamura, A. Matsumoto, A. E. Eltayeb, H. Tsujimoto, Preferential recruitment of the maternal centromere-specific histone H3 (CENH3) in oat (*Avena sativa* L.) × pearl millet (*Pennisetum glaucum* L.) hybrid embryos. Chromosome Res. 23, 709–718 (2015). 26134441 10.1007/s10577-015-9477-5

[13] M. Ingouff, S. Rademacher, S. Holec, L. Soljić, N. Xin, A. Readshaw, S. H. Foo, B. Lahouze, S. Sprunck, F. Berger, Zygotic resetting of the HISTONE 3 variant repertoire participates in epigenetic reprogramming in Arabidopsis. Curr. Biol. 20, 2137–2143 (2010). 21093266 10.1016/j.cub.2010.11.012

[14] D. Maruyama, Y. Hamamura, H. Takeuchi, D. Susaki, M. Nishimaki, D. Kurihara, R. D. Kasahara, T. Higashiyama, Independent control by each female gamete prevents the attraction of multiple pollen tubes. Dev. Cell 25, 317–323 (2013). 23673333 10.1016/j.devcel.2013.03.013

[15] D. Carputo, L. Monti, J. E. Werner, L. Frusciante, Uses and usefulness of endosperm balance number. Theor. Appl. Genet. 98, 478–484 (1999).

[16] H.-y. Zhang, M. Luo, S. D. Johnson, X.-w. Zhu, L. Liu, F. Huang, Y.-t. Liu, P.-z. Xu, X.-j. Wu, Parental genome imbalance causes post-zygotic seed lethality and deregulates imprinting in rice. Rice 9, 43 (2016). 27568375 10.1186/s12284-016-0115-4 PMC5002275

[17] M. Ravi, M. P. A. Marimuthu, E. H. Tan, S. Maheshwari, I. M. Henry, B. Marin-Rodriguez, G. Urtecho, J. Tan, K. Thornhill, F. Zhu, A. Panoli, V. Sundaresan, A. B. Britt, L. Comai, S. W. L. Chan, A haploid genetics toolbox for Arabidopsis thaliana. Nat. Commun. 5, 5334 (2014). 25358957 10.1038/ncomms6334

[18] K. Gooh, M. Ueda, K. Aruga, J. Park, H. Arata, T. Higashiyama, D. Kurihara, Live-cell imaging and optical manipulation of Arabidopsis early embryogenesis. Dev. Cell 34, 242–251 (2015). 26166301 10.1016/j.devcel.2015.06.008

[19] M. Ingouff, Y. Hamamura, M. Gourgues, T. Higashiyama, F. Berger, Distinct dynamics of HISTONE3 variants between the two fertilization products in plants. Curr. Biol. 17, 1032–1037 (2007). 17555967 10.1016/j.cub.2007.05.019

[20] R. J. Scott, M. Spielman, J. Bailey, H. G. Dickinson, Parent-of-origin effects on seed development in Arabidopsis thaliana. Development 125, 3329–3341 (1998). 9693137 10.1242/dev.125.17.3329

[21] I. M. Henry, B. P. Dilkes, K. Young, B. Watson, H. Wu, L. Comai, Aneuploidy and genetic variation in the Arabidopsis thaliana triploid response. Genetics 170, 1979–1988 (2005). 15944363 10.1534/genetics.104.037788 PMC1449780

[22] M. P. A. Marimuthu, S. Jolivet, M. Ravi, L. Pereira, J. N. Davda, L. Cromer, L. Wang, F. Nogué, S. W. L. Chan, I. Siddiqi, R. Mercier, Synthetic clonal reproduction through seeds. Science 331, 876 (2011). 21330535 10.1126/science.1199682

[23] P. B. Talbert, R. Masuelli, A. P. Tyagi, L. Comai, S. Henikoff, Centromeric localization and adaptive evolution of an Arabidopsis histone H3 variant. Plant Cell 14, 1053–1066 (2002). 12034896 10.1105/tpc.010425 PMC150606

[24] S. Maheshwari, T. Ishii, C. T. Brown, A. Houben, L. Comai, Centromere location in Arabidopsis is unaltered by extreme divergence in CENH3 protein sequence. Genome Res. 27, 471–478 (2017). 28223399 10.1101/gr.214619.116 PMC5340974

[25] T. Ishii, M. Juranić, S. Maheshwari, F. de O. Bustamante, M. Vogt, R. Salinas-Gamboa, S. Dreissig, N. Gursanscky, T. How, D. Demidov, J. Fuchs, V. Schubert, A. Spriggs, J.-P. Vielle-Calzada, L. Comai, A. M. G. Koltunow, A. Houben, Unequal contribution of two paralogous CENH3 variants in cowpea centromere function. Commun. Biol. 3, 775 (2020). 33319863 10.1038/s42003-020-01507-x PMC7738545

[26] A. Houben, T. Wako, R. Furushima-Shimogawara, G. Presting, G. Kunzel, I. Schubert, I. K. Fukui, Short communication: The cell cycle dependent phosphorylation of histone H3 is correlated with the condensation of plant mitotic chromosomes. Plant J. 18, 675–679 (1999). 10417719 10.1046/j.1365-313x.1999.00496.x

[27] E. Kaszás, W. Z. Cande, Phosphorylation of histone H3 is correlated with changes in the maintenance of sister chromatid cohesion during meiosis in maize, rather than the condensation of the chromatin. J. Cell Sci. 113 ( Pt. 18), 3217–3226 (2000). 10954420 10.1242/jcs.113.18.3217

[28] F. Shibata, M. Murata, Differential localization of the centromere-specific proteins in the major centromeric satellite of Arabidopsis thaliana. J. Cell Sci. 117, 2963–2970 (2004). 15161939 10.1242/jcs.01144

[29] A. Houben, D. Demidov, A. D. Caperta, R. Karimi, F. Agueci, L. Vlasenko, Phosphorylation of histone H3 in plants—A dynamic affair. Biochim. Biophys. Acta 1769, 308–315 (2007). 17320987 10.1016/j.bbaexp.2007.01.002

[30] B. A. Sullivan, G. H. Karpen, Centromeric chromatin exhibits a histone modification pattern that is distinct from both euchromatin and heterochromatin. Nat. Struct. Mol. Biol. 11, 1076–1083 (2004). 15475964 10.1038/nsmb845 PMC1283111

[31] W. Zhang, H.-R. Lee, D.-H. Koo, J. Jiang, Epigenetic modification of centromeric chromatin: Hypomethylation of DNA sequences in the CENH3-associated chromatin in Arabidopsis thaliana and maize. Plant Cell 20, 25–34 (2008). 18239133 10.1105/tpc.107.057083 PMC2254920

[32] A. Musacchio, A. Desai, A molecular view of kinetochore assembly and function. Biology 6, 5 (2017). 28125021 10.3390/biology6010005 PMC5371998

[33] D. Gernand, T. Rutten, A. Varshney, M. Rubtsova, S. Prodanovic, C. Bruss, J. Kumlehn, F. Matzk, A. Houben, Uniparental chromosome elimination at mitosis and interphase in wheat and pearl millet crosses involves micronucleus formation, progressive heterochromatinization, and DNA fragmentation. Plant Cell 17, 2431–2438 (2005). 16055632 10.1105/tpc.105.034249 PMC1197425

[34] P. Ly, L. S. Teitz, D. H. Kim, O. Shoshani, H. Skaletsky, D. Fachinetti, D. C. Page, D. W. Cleveland, Selective Y centromere inactivation triggers chromosome shattering in micronuclei and repair by non-homologous end joining. Nat. Cell Biol. 19, 68–75 (2017). 27918550 10.1038/ncb3450 PMC5539760

[35] M. L. Leibowitz, C.-Z. Zhang, D. Pellman, Chromothripsis: A new mechanism for rapid karyotype evolution. Annu. Rev. Genet. 49, 183–211 (2015). 26442848 10.1146/annurev-genet-120213-092228

[36] T. Ishii, T. Ueda, H. Tanaka, H. Tsujimoto, Chromosome elimination by wide hybridization between Triticeae or oat plant and pearl millet: Pearl millet chromosome dynamics in hybrid embryo cells. Chromosome Res. 18, 821–831 (2010). 20953694 10.1007/s10577-010-9158-3

[37] R. Gibeaux, R. Acker, M. Kitaoka, G. Georgiou, I. van Kruijsbergen, B. Ford, E. M. Marcotte, D. K. Nomura, T. Kwon, G. J. C. Veenstra, R. Heald, Paternal chromosome loss and metabolic crisis contribute to hybrid inviability in Xenopus. Nature 553, 337–341 (2018). 29320479 10.1038/nature25188 PMC5988642

[38] A. Fujiwara, S. Abe, E. Yamaha, F. Yamazaki, M. C. Yoshida, Uniparental chromosome elimination in the early embryogenesis of the inviable salmonid hybrids between masu salmon female and rainbow trout male. Chromosoma 106, 44–52 (1997). 9169586 10.1007/s004120050223

[39] E. H. Tan, I. M. Henry, M. Ravi, K. R. Bradnam, T. Mandakova, M. P. Marimuthu, I. Korf, M. A. Lysak, L. Comai, S. W. Chan, Catastrophic chromosomal restructuring during genome elimination in plants. eLife 4, e06516 (2015). 25977984 10.7554/eLife.06516 PMC4461816

[40] M. Ravi, F. Shibata, J. S. Ramahi, K. Nagaki, C. Chen, M. Murata, S. W. Chan, Meiosis-specific loading of the centromere-specific histone CENH3 in *Arabidopsis thaliana*. PLOS Genet. 7, e1002121 (2011). 21695238 10.1371/journal.pgen.1002121 PMC3111537

[41] M. Borg, Y. Jacob, D. Susaki, C. LeBlanc, D. Buendía, E. Axelsson, T. Kawashima, P. Voigt, L. Boavida, J. Becker, T. Higashiyama, R. Martienssen, F. Berger, Targeted reprogramming of H3K27me3 resets epigenetic memory in plant paternal chromatin. Nat. Cell Biol. 22, 621–629 (2020). 32393884 10.1038/s41556-020-0515-y PMC7116658

[42] Z. Mérai, N. Chumak, M. García-Aguilar, T.-F. Hsieh, T. Nishimura, V. K. Schoft, J. Bindics, L. Slusarz, S. Arnoux, S. Opravil, K. Mechtler, D. Zilberman, R. L. Fischer, H. Tamaru, The AAA-ATPase molecular chaperone Cdc48/p97 disassembles sumoylated centromeres, decondenses heterochromatin, and activates ribosomal RNA genes. Proc. Natl. Acad. Sci. U.S.A. 111, 16166–16171 (2014). 25344531 10.1073/pnas.1418564111 PMC4234600

[43] S. Kuppu, E. H. Tan, H. Nguyen, A. Rodgers, L. Comai, S. W. L. Chan, A. B. Britt, Point mutations in centromeric histone induce post-zygotic incompatibility and uniparental inheritance. PLOS Genet. 11, e1005494 (2015). 26352591 10.1371/journal.pgen.1005494 PMC4564284

[44] N. Raychaudhuri, R. Dubruille, G. A. Orsi, H. C. Bagheri, B. Loppin, C. F. Lehner, Transgenerational propagation and quantitative maintenance of paternal centromeres depends on Cid/Cenp-A presence in Drosophila sperm. PLOS Biol. 10, e1001434 (2012). 23300376 10.1371/journal.pbio.1001434 PMC3531477

[45] M. Ravi, P. N. Kwong, R. M. G. Menorca, J. T. Valencia, J. S. Ramahi, J. L. Stewart, R. K. Tran, V. Sundaresan, L. Comai, S. W.-L. Chan, The rapidly evolving centromere-specific histone has stringent functional requirements in Arabidopsis thaliana. Genetics 186, 461–471 (2010). 20628040 10.1534/genetics.110.120337 PMC2954480

[46] R. Gassmann, A. Rechtsteiner, K. W. Yuen, A. Muroyama, T. Egelhofer, L. Gaydos, F. Barron, P. Maddox, A. Essex, J. Monen, S. Ercan, J. D. Lieb, K. Oegema, S. Strome, A. Desai, An inverse relationship to germline transcription defines centromeric chromatin in *C. elegans*. Nature 484, 534–537 (2012). 22495302 10.1038/nature10973 PMC3538161

[47] S. Mitra, D. L. Bodor, A. F. David, I. Abdul-Zani, J. F. Mata, B. Neumann, S. Reither, C. Tischer, L. E. T. Jansen, Genetic screening identifies a SUMO protease dynamically maintaining centromeric chromatin. Nat. Commun. 11, 501 (2020). 31980633 10.1038/s41467-019-14276-x PMC6981222

[48] H. R. Woo, O. Pontes, C. S. Pikaard, E. J. Richards, VIM1, a methylcytosine-binding protein required for centromeric heterochromatinization. Genes Dev. 21, 267–277 (2007). 17242155 10.1101/gad.1512007 PMC1785122

[49] E. Kraft, M. Bostick, S. E. Jacobsen, J. Callis, ORTH/VIM proteins that regulate DNA methylation are functional ubiquitin E3 ligases. Plant J. 56, 704–715 (2008). 18643997 10.1111/j.1365-313X.2008.03631.x PMC2973330

[50] L. M. Johnson, M. Bostick, X. Zhang, E. Kraft, I. Henderson, J. Callis, S. E. Jacobsen, The SRA methyl-cytosine-binding domain links DNA and histone methylation. Curr. Biol. 17, 379–384 (2007). 17239600 10.1016/j.cub.2007.01.009 PMC1850948

[51] G. Hewawasam, M. Shivaraju, M. Mattingly, S. Venkatesh, S. Martin-Brown, L. Florens, J. L. Workman, J. L. Gerton, Psh1 is an E3 ubiquitin ligase that targets the centromeric histone variant Cse4. Mol. Cell 40, 444–454 (2010). 21070970 10.1016/j.molcel.2010.10.014 PMC2998187

[52] P. Ranjitkar, M. O. Press, X. Yi, R. Baker, M. J. MacCoss, S. Biggins, An E3 ubiquitin ligase prevents ectopic localization of the centromeric histone H3 variant via the centromere targeting domain. Mol. Cell 40, 455–464 (2010). 21070971 10.1016/j.molcel.2010.09.025 PMC2995698

[53] Y. Niikura, R. Kitagawa, L. Fang, K. Kitagawa, CENP-A ubiquitylation is indispensable to cell viability. Dev. Cell 50, 683–689.e6 (2019). 31550462 10.1016/j.devcel.2019.07.015 PMC6761987

[54] D. L. Bodor, J. F. Mata, M. Sergeev, A. F. David, K. J. Salimian, T. Panchenko, D. W. Cleveland, B. E. Black, J. V. Shah, L. E. Jansen, The quantitative architecture of centromeric chromatin. eLife 3, e02137 (2014). 25027692 10.7554/eLife.02137 PMC4091408

[55] S. Henikoff, Dosage-dependent modification of position-effect variegation in Drosophla. Bioessays 18, 401–409 (1996). 8639163 10.1002/bies.950180510

[56] V. Pirrotta, L. Rastelli, White gene expression, repressive chromatin domains and homeotic gene regulation in Drosophila. Bioessays 16, 549–556 (1994). 7916186 10.1002/bies.950160808

[57] A. TenHarmsel, R. J. Austin, N. Savenelli, M. D. Biggin, Cooperative binding at a distance by even-skipped protein correlates with repression and suggests a mechanism of silencing. Mol. Cell. Biol. 13, 2742–2752 (1993). 8097276 10.1128/mcb.13.5.2742-2752.1993 PMC359652

[58] N. Wang, R. K. Dawe, Centromere size and its relationship to haploid formation in plants. Mol. Plant 11, 398–406 (2018). 29277426 10.1016/j.molp.2017.12.009

[59] Y. Niikura, R. Kitagawa, K. Kitagawa, CENP-A ubiquitylation contributes to maintaining the chromosomal location of the centromere. Molecules 24, 402 (2019). 30678315 10.3390/molecules24030402 PMC6384693

[60] S. Henikoff, K. Ahmad, H. S. Malik, The centromere paradox: Stable inheritance with rapidly evolving DNA. Science 293, 1098–1102 (2001). 11498581 10.1126/science.1062939

[61] L. Rosin, B. G. Mellone, Co-evolving CENP-A and CAL1 domains mediate centromeric CENP-A deposition across Drosophila species. Dev. Cell 37, 136–147 (2016). 27093083 10.1016/j.devcel.2016.03.021 PMC4861639

[62] I. A. Drinnenberg, S. Henikoff, H. S. Malik, Evolutionary turnover of kinetochore proteins: A ship of theseus? Trends Cell Biol. 26, 498–510 (2016). 26877204 10.1016/j.tcb.2016.01.005 PMC4914419

[63] L. F. Rosin, B. G. Mellone, Centromeres drive a hard bargain. Trends Genet. 33, 101–117 (2017). 28069312 10.1016/j.tig.2016.12.001 PMC5467322

[64] K. Ohkuni, Y. Takahashi, A. Fulp, J. Lawrimore, W.-C. Au, N. Pasupala, R. Levy-Myers, J. Warren, A. Strunnikov, R. E. Baker, O. Kerscher, K. Bloom, M. A. Basrai, SUMO-targeted ubiquitin ligase (STUbL) Slx5 regulates proteolysis of centromeric histone H3 variant Cse4 and prevents its mislocalization to euchromatin. Mol. Biol. Cell 27, 1500–1510 (2016). 26960795 10.1091/mbc.E15-12-0827 PMC4850037

[65] X. Zhang, R. Henriques, S.-S. Lin, Q.-W. Niu, N.-H. Chua, Agrobacterium-mediated transformation of Arabidopsis thaliana using the floral dip method. Nat. Protoc. 1, 641–646 (2006). 17406292 10.1038/nprot.2006.97

[66] H. Tsutsui, T. Higashiyama, pKAMA-ITACHI vectors for highly efficient CRISPR/Cas9-mediated gene knockout in Arabidopsis thaliana. Plant Cell Physiol. 58, 46–56 (2017). 27856772 10.1093/pcp/pcw191 PMC5444565

[67] D. R. Smyth, J. L. Bowman, E. M. Meyerowitz, Early flower development in Arabidopsis. Plant Cell 2, 755–767 (1990). 2152125 10.1105/tpc.2.8.755 PMC159928

[68] K. Schneitz, M. Hülskamp, R. E. Pruitt, Wild-type ovule development in Arabidopsis thaliana: A light microscope study of cleared whole-mount tissue. Plant J. 7, 731–749 (1995).

[69] W. She, C. Baroux, Chromatin dynamics during plant sexual reproduction. Front. Plant Sci. 5, 354 (2014). 25104954 10.3389/fpls.2014.00354 PMC4109563

[70] V. Sundaresan, P. Springer, T. Volpe, S. Haward, J. D. Jones, C. Dean, H. Ma, R. Martienssen, Patterns of gene action in plant development revealed by enhancer trap and gene trap transposable elements. Genes Dev. 9, 1797–1810 (1995). 7622040 10.1101/gad.9.14.1797

[71] T. Kinoshita, A. Miura, Y. Choi, Y. Kinoshita, X. Cao, S. E. Jacobsen, R. L. Fischer, T. Kakutani, One-way control of FWA imprinting in Arabidopsis endosperm by DNA methylation. Science 303, 521–523 (2004). 14631047 10.1126/science.1089835

[72] A. P. Joglekar, E. D. Salmon, K. S. Bloom, in *Methods in Cell Biology* (Academic Press, 2008), vol. 85, pp. 127–151; www.sciencedirect.com/science/article/pii/S0091679X08850078. 10.1016/S0091-679X(08)85007-8 PMC2892121 18155462

[73] M. Ravi, R. Bondada, Genome elimination by Tailswap CenH3: In vivo haploid production in *Arabidopsis thaliana*, in *Chromosome and Genomic Engineering in Plants: Methods and Protocols*, M. Murata, Ed. (Springer, 2016), pp. 77–99; 10.1007/978-1-4939-4931-1_6. 27557687

[74] M. D. Blower, B. A. Sullivan, G. H. Karpen, Conserved organization of centromeric chromatin in flies and humans. Dev. Cell 2, 319–330 (2002). 11879637 10.1016/s1534-5807(02)00135-1 PMC3192492

