# Supplementary material for: Epigenetically mismatched parental centromeres trigger genome elimination in hybrids
Source: Sci Adv. 2021 Nov 19;7(47):eabk1151. doi: 10.1126/sciadv.abk1151 (PMC8604413; doi:10.1126/sciadv.abk1151)
Supplement: Supplementary file 1 — Figs. S1 to S9 [file sciadv.abk1151_sm.pdf]

Science Advances
AAAS

Supplementary Materials for

Epigenetically mismatched parental centromeres trigger genome elimination in hybrids

Mohan P. A. Marimuthu, Ravi Maruthachalam, Ramesh Bondada, Sundaram Kuppu, Ek Han Tan, Anne Britt, Simon W. L. Chan, Luca Comai*

*Corresponding author. Email: lcomai@ucdavis.edu

Published 19 November 2021, *Sci. Adv.* **7**, eabk1151 (2021)
DOI: 10.1126/sciadv.abk1151

This PDF file includes:

Figs. S1 to S9

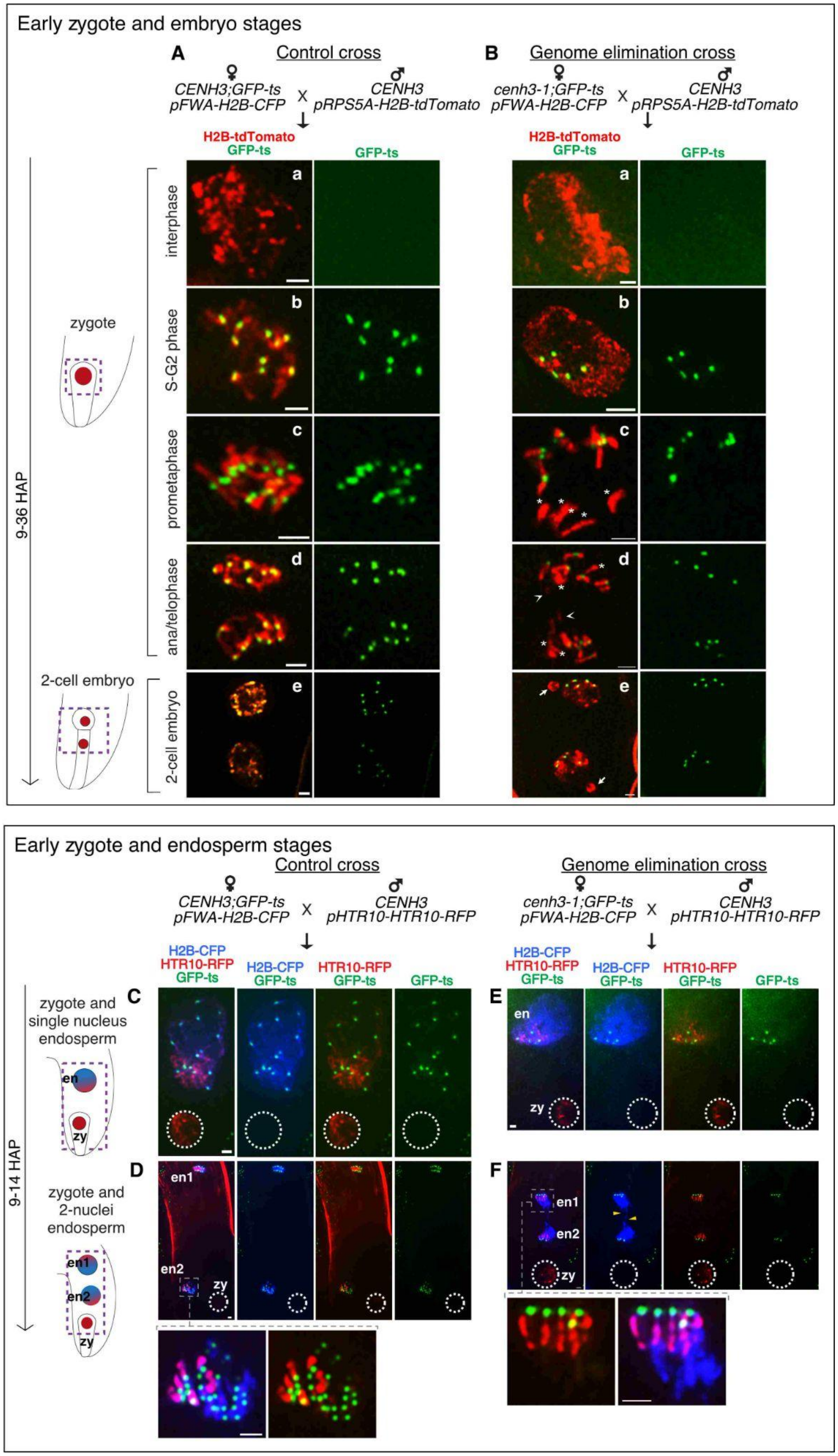

Early zygote and embryo stages
A
Control cross
B
Genome elimination cross
CENH3;GFP-ts
pFWA-H2B-CFP
X
CENH3
pRPS5A-H2B-tdTomato
cenh3-1;GFP-ts
pFWA-H2B-CFP
X
CENH3
pRPS5A-H2B-tdTomato
H2B-tdTomato
GFP-ts
GFP-ts
interphase
S-G2 phase
prometaphase
ana/telophase
2-cell embryo
zygote
2-cell embryo
9-36 HAP
Early zygote and endosperm stages
Control cross
Genome elimination cross
CENH3;GFP-ts
pFWA-H2B-CFP
X
CENH3
pHTR10-HTR10-RFP
cenh3-1;GFP-ts
pFWA-H2B-CFP
X
CENH3
pHTR10-HTR10-RFP
H2B-CFP
HTR10-RFP
GFP-ts
H2B-CFP
GFP-ts
HTR10-RFP
GFP-ts
GFP-ts
C
D
E
F
zygote and
single nucleus
endosperm
zygote and
2-nuclei
endosperm
9-14 HAP
en
zy
en1
en2

Fig. S1. Uniparental localization of maternal GFP-ts during haploid induction. The ovule schematic on the left indicates the region of interest in the ovule shown on the right (**A to F**). Progressive stages of zygotic development in CC (**A-a** to **A-e**) and GEC (**B-a** to **B-e**) captured from multiple ovules. "*" marks condensed chromosomes without centromeric GFP-ts (**B-c**, **B-d**); arrowheads: lagging chromosomes (**B-d**); arrows: micronuclei (**B-e**). Endosperm nuclei displaying male (red) and biparental (blue) chromatin in interphase (**C, E**) and metaphase (**D**) or anaphase chromatin (**F**). Yellow triangles: female chromatin bridge (**F**). White dotted circles: Zygote (**C to F**). Part of this material was used for the data shown in main figure 1E to 1I. en: endosperm; zy: zygote; HAP: hours after pollination. Scale bar = 1 μm.

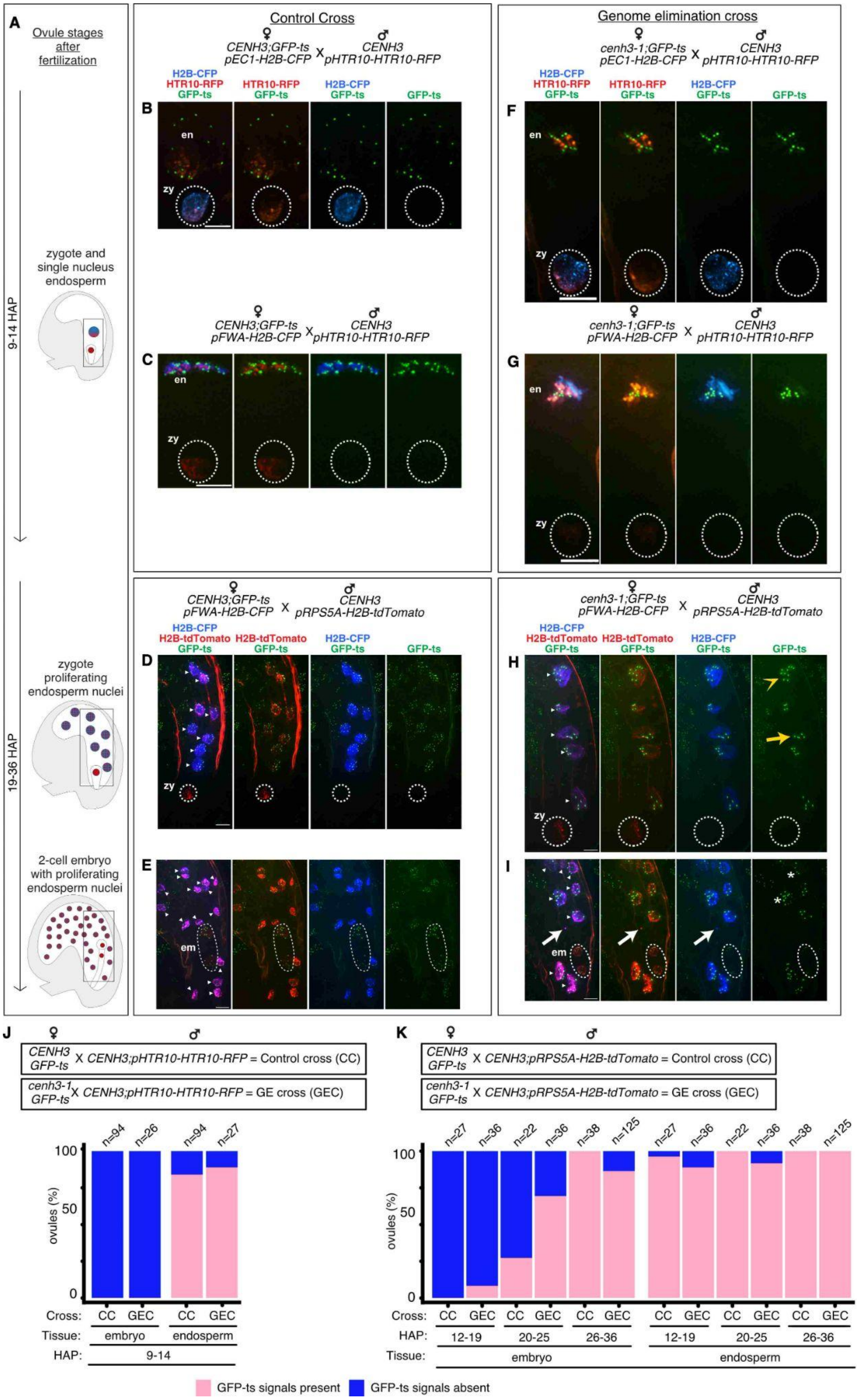
A
Ovule stages after fertilization
9-14 HAP
zygote and single nucleus endosperm
19-36 HAP
zygote proliferating endosperm nuclei
2-cell embryo with proliferating endosperm nuclei
Control Cross
CENH3;GFP-ts pEC1-H2B-CFP X CENH3 pHTR10-HTR10-RFP
CENH3;GFP-ts pFWA-H2B-CFP X CENH3 pHTR10-HTR10-RFP
CENH3;GFP-ts pFWA-H2B-CFP X CENH3 pRPS5A-H2B-tdTomato
Genome elimination cross
cenh3-1;GFP-ts pEC1-H2B-CFP X CENH3 pHTR10-HTR10-RFP
cenh3-1;GFP-ts pFWA-H2B-CFP X CENH3 pHTR10-HTR10-RFP
cenh3-1;GFP-ts pFWA-H2B-CFP X CENH3 pRPS5A-H2B-tdTomato
H2B-CFP HTR10-RFP GFP-ts
H2B-CFP H2B-tdTomato GFP-ts
B C D E F G H I
en zy em
J
CENH3 GFP-ts X CENH3;pHTR10-HTR10-RFP = Control cross (CC)
cenh3-1 GFP-ts X CENH3;pHTR10-HTR10-RFP = GE cross (GEC)
n=94 n=26 n=94 n=27
ovules (%)
Cross: CC GEC CC GEC
Tissue: embryo endosperm
HAP: 9-14
K
CENH3 GFP-ts X CENH3;pRPS5A-H2B-tdTomato = Control cross (CC)
cenh3-1 GFP-ts X CENH3;pRPS5A-H2B-tdTomato = GE cross (GEC)
n=27 n=36 n=22 n=36 n=38 n=125 n=27 n=36 n=22 n=36 n=38 n=125
HAP: 12-19 20-25 26-36 12-19 20-25 26-36
Tissue: embryo endosperm
GFP-ts signals present
GFP-ts signals absent

Fig. S2. Haploid induction is marked by the presence of maternal GFP-ts on one of the parental chromatin sets (x=5). (**A**) Schematic representation depicting the region of interest in the ovule stages shown in the CC (**B to E**) and GEC (**F to I**) images on the right. Lines expressing *pHTR10-RFP* (a sperm-specific histone H3.3 variant marker in wild-type background) are used as male parent in **B, C, F** and **G** whereas lines expressing *pRPS5A-H2B-tdTomato* (in wild-type background), a constitutive marker, are used as male parent in **D, E, H** and **I**. Zygote (zy) and embryo (em) are marked with dotted white circles and ellipses, respectively. Endosperm is marked by "en" or solid white triangles. (**H**) The yellow arrowhead and yellow arrow mark endosperm nuclei with 10 and 5 centromeric GFP-ts signals respectively . (**I**) "*" marks the endosperm nuclei carrying bright and fainter centromeric GFP-ts signals. White arrow marks micronuclei. Quantification of presence and absence of GFP-ts signals in zygote and endosperm nuclei at early stages of development in control and genome elimination crosses with *pHTR10-HTR10-RFP* (**J**) or *pRPS5A-H2B-tdTomato* (**K**) as male parent. n=number of ovules. HAP: hours after pollination. Scale bar = 5µm.

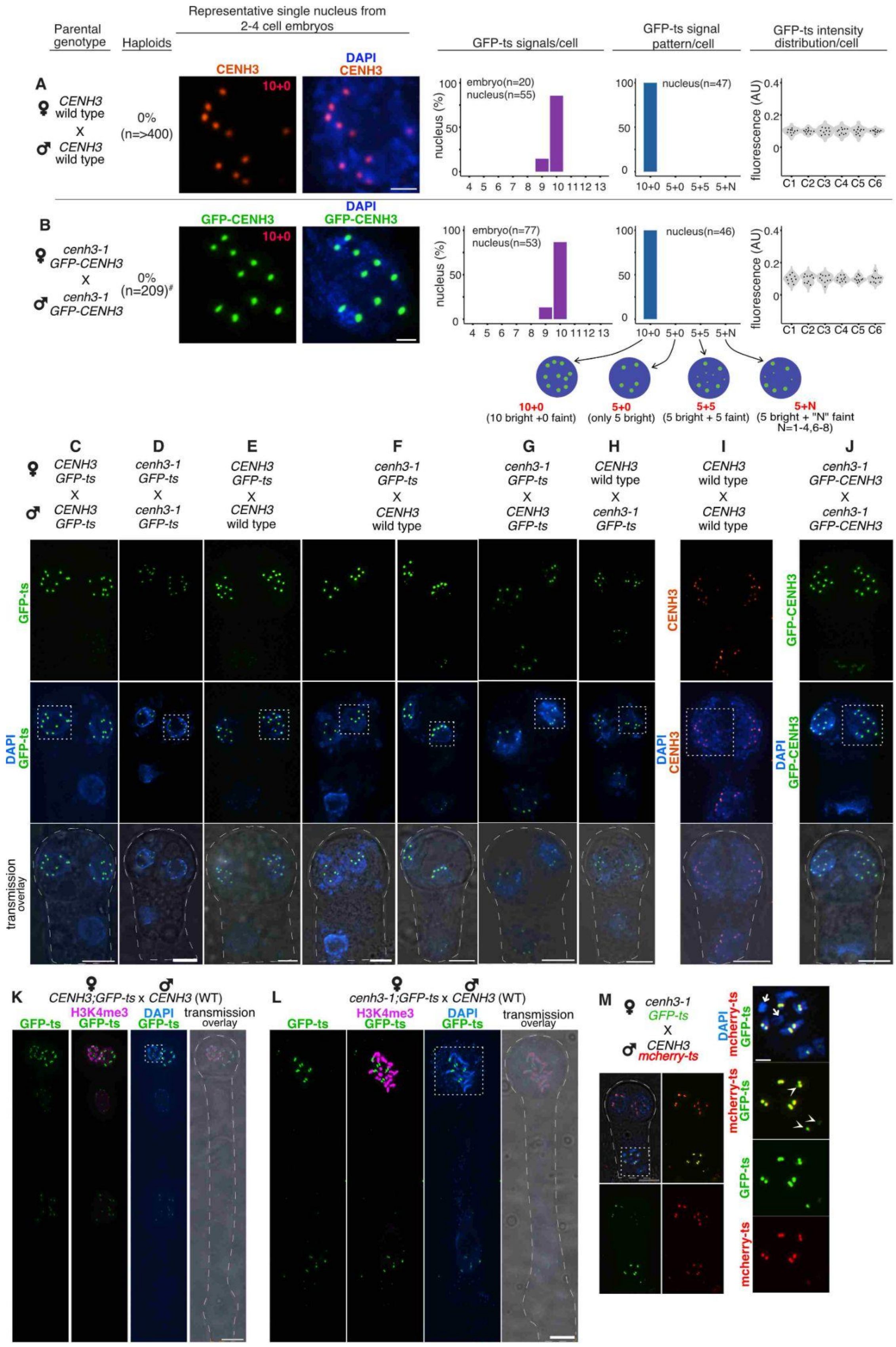
Parental genotype
Haploids
Representative single nucleus from 2-4 cell embryos
GFP-ts signals/cell
GFP-ts signal pattern/cell
GFP-ts intensity distribution/cell
A
♀ CENH3 wild type X ♂ CENH3 wild type
0% (n=>400)
CENH3
DAPI CENH3
10+0
embryo(n=20) nucleus(n=55)
nucleus(n=47)
nucleus (%)
fluorescence (AU)
B
♀ cenh3-1 GFP-CENH3 X ♂ cenh3-1 GFP-CENH3
0% (n=209)#
GFP-CENH3
DAPI GFP-CENH3
embryo(n=77) nucleus(n=53)
nucleus(n=46)
10+0 (10 bright +0 faint)
5+0 (only 5 bright)
5+5 (5 bright + 5 faint)
5+N (5 bright + "N" faint N=1-4,6-8)
C ♀ CENH3 GFP-ts X ♂ CENH3 GFP-ts
D cenh3-1 GFP-ts X cenh3-1 GFP-ts
E CENH3 GFP-ts X CENH3 wild type
F cenh3-1 GFP-ts X CENH3 wild type
G cenh3-1 GFP-ts X CENH3 GFP-ts
H CENH3 wild type X cenh3-1 GFP-ts
I CENH3 wild type X CENH3 wild type
J cenh3-1 GFP-CENH3 X cenh3-1 GFP-CENH3
GFP-ts
DAPI GFP-ts
transmission overlay
CENH3
DAPI CENH3
GFP-CENH3
DAPI GFP-CENH3
K ♀ CENH3;GFP-ts x ♂ CENH3 (WT)
L ♀ cenh3-1;GFP-ts x ♂ CENH3 (WT)
H3K4me3 GFP-ts
DAPI GFP-ts
M ♀ cenh3-1 GFP-ts X ♂ CENH3 mcherry-ts
DAPI mcherry-ts GFP-ts
mcherry-ts GFP-ts
mcherry-ts

Fig. S3. Biased loading of GFP-ts onto one set(x=5) of the parental centromeres at 2-4 cell stage hybrid embryos highlights genome elimination.

Embryo nuclei from progeny of selfed (**A**) wild-type (CENH3 immunolocalization in red) and selfed (**B**) *cenh3-1* mutant complemented with the GFP-tagged-CENH3 construct (GFP-CENH3). For the displayed images, corresponding patterns of CENH3 (**A**) or CENH3-GFP (**B**) signals are marked, respectively, on the red and green channel for each genotype.The bar graphs on the right display quantification for the number of GFP-ts signals, and pattern per cell for each cross shown on the left. Each column in the violin plot indicates relative GFP-ts signal intensity in arbitrary units(AU) within a single cell (C1-C6). (**C to L**) A part of this material (white dotted box) is used for the single nuclei data presented in Fig. 3, A to I and **A** and **B** in this figure. (**M**) Paternal *mcherry-ts* reflects the maternal GFP-ts loading pattern in a HI cross. White dotted area is magnified on the right highlighting the pre-mitotic stage. White arrowheads mark faint signals; white arrows mark condensed chromosomes without any centromeric signals. "#" Data from (Ravi and Chan, 2010). Scale bar = 1 μm(for single nucleus); 5 μm (for whole embryos).

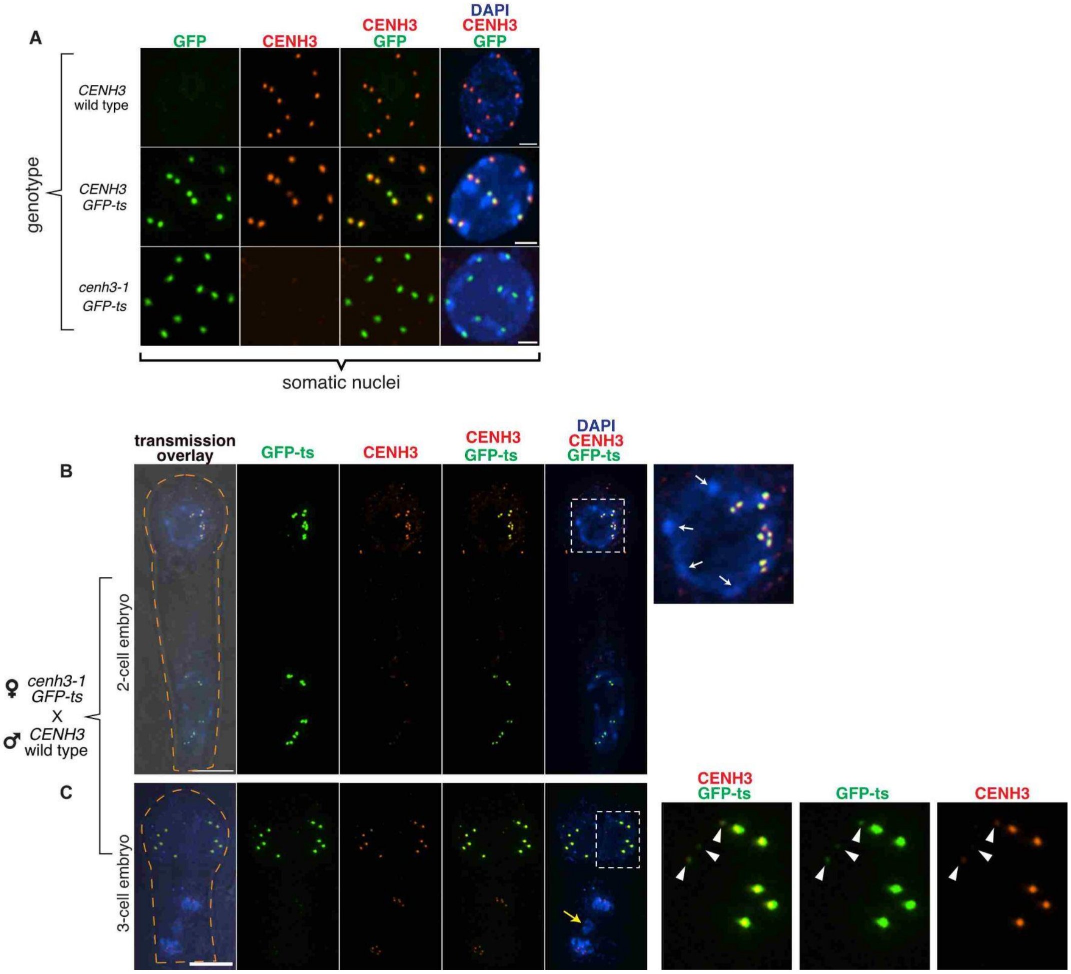

Fig. S4. Wild-type CENH3 and GFP-ts occupy the same functional centromeres in embryos undergoing GE.

(**A**) Representative somatic cell nuclear staining with anti-CENH3 antibody demonstrating the absence of cross-reactivity with the GFP-ts variant. (**B,C**) A representative nucleus (white dotted box) from the embryos **B** and **C** shown on the left are zoomed on the right of the respective image rows. Colocalization of GFP-ts and CENH3 on one parental set of centromeres in G2 nucleus of 2-cell (**B**) and interphase nuclei of 3-cell (**C**) stage embryo from GE cross. White arrows mark chromocenters without CENH3 or GFP-ts signals. Yellow arrow indicates laggard chromosomes without CENH3 or GFP-ts signals. White triangle shows colocalization of faint CENH3 signals with the faint GFP-ts signals. Scale bar = 5μm for the whole embryo images and 1 μm for the rest of the figure.

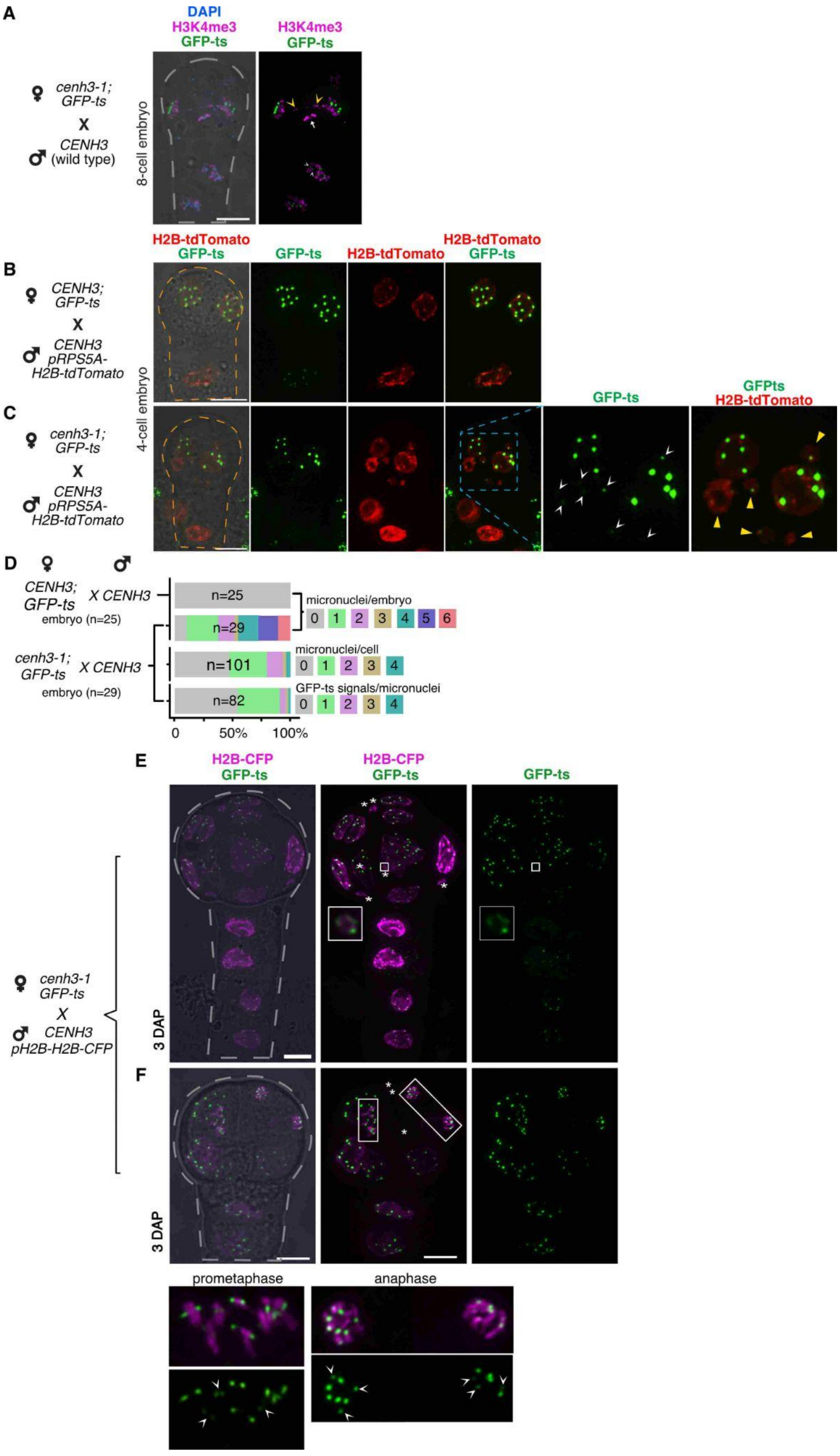

A
DAPI
H3K4me3
GFP-ts
H3K4me3
GFP-ts
♀ cenh3-1; GFP-ts
X
♂ CENH3 (wild type)
8-cell embryo
B
H2B-tdTomato
GFP-ts
GFP-ts
H2B-tdTomato
H2B-tdTomato
GFP-ts
♀ CENH3; GFP-ts
X
♂ CENH3 pRPS5A-H2B-tdTomato
4-cell embryo
C
♀ cenh3-1; GFP-ts
X
♂ CENH3 pRPS5A-H2B-tdTomato
GFP-ts
GFPts
H2B-tdTomato
D ♀ ♂
CENH3; GFP-ts X CENH3
embryo (n=25)
cenh3-1; GFP-ts X CENH3
embryo (n=29)
n=25
n=29
n=101
n=82
micronuclei/embryo
0 1 2 3 4 5 6
micronuclei/cell
0 1 2 3 4
GFP-ts signals/micronuclei
0 1 2 3 4
0 50% 100%
E
H2B-CFP
GFP-ts
H2B-CFP
GFP-ts
GFP-ts
♀ cenh3-1 GFP-ts
X
♂ CENH3 pH2B-H2B-CFP
3 DAP
F
3 DAP
prometaphase
anaphase

Fig. S5. Fate of HI chromosomes in developing embryos.

Early embryonic mitosis in GE (**A, C, E** and **F**) and control crosses (**B**). (**A**) H3K4me3 staining highlights the unstable HI parent chromosomes from an 8-cell stage embryo (only partial z-sections are shown). Yellow arrowhead: chromatin bridge; white arrowheads: faint GFP-ts signal on normally segregating chromosomes; white arrow: laggard chromosomes. 4-cell stage of embryos in control (**B**) and GE cross (**C**) with nucleus marked by H2B-tdTomato. (**C**, right, selected region of interest highlighted in dotted blue box on the embryo) Micronuclei (yellow triangles) containing weakly labeled centromeres of HI (white arrowheads). (**D**) The bar graph depicts a quantitative analysis of micronuclei and GFP-ts signals in control and HI crosses represented in figures **B** and **C**. (**E**) Embryo from an GE cross from ovules 3 DAP showing the presence of multiple micronuclei (marked by "*"). Enhanced inset: micronuclei with faint GFP-ts signal. (**F**) Embryo containing multiple micronuclei (marked by "*") with prometaphase and anaphase stage cells highlighted (rectangle). Note that both prometaphase and anaphase cells display bright and faint GFP-ts centromeric signals. The chromosomes with fainter signals(white arrowheads on the bottom right) appear to segregate normally along with chromosomes with brighter signals. DAP: Days after pollination. Scale bar = 5μm.

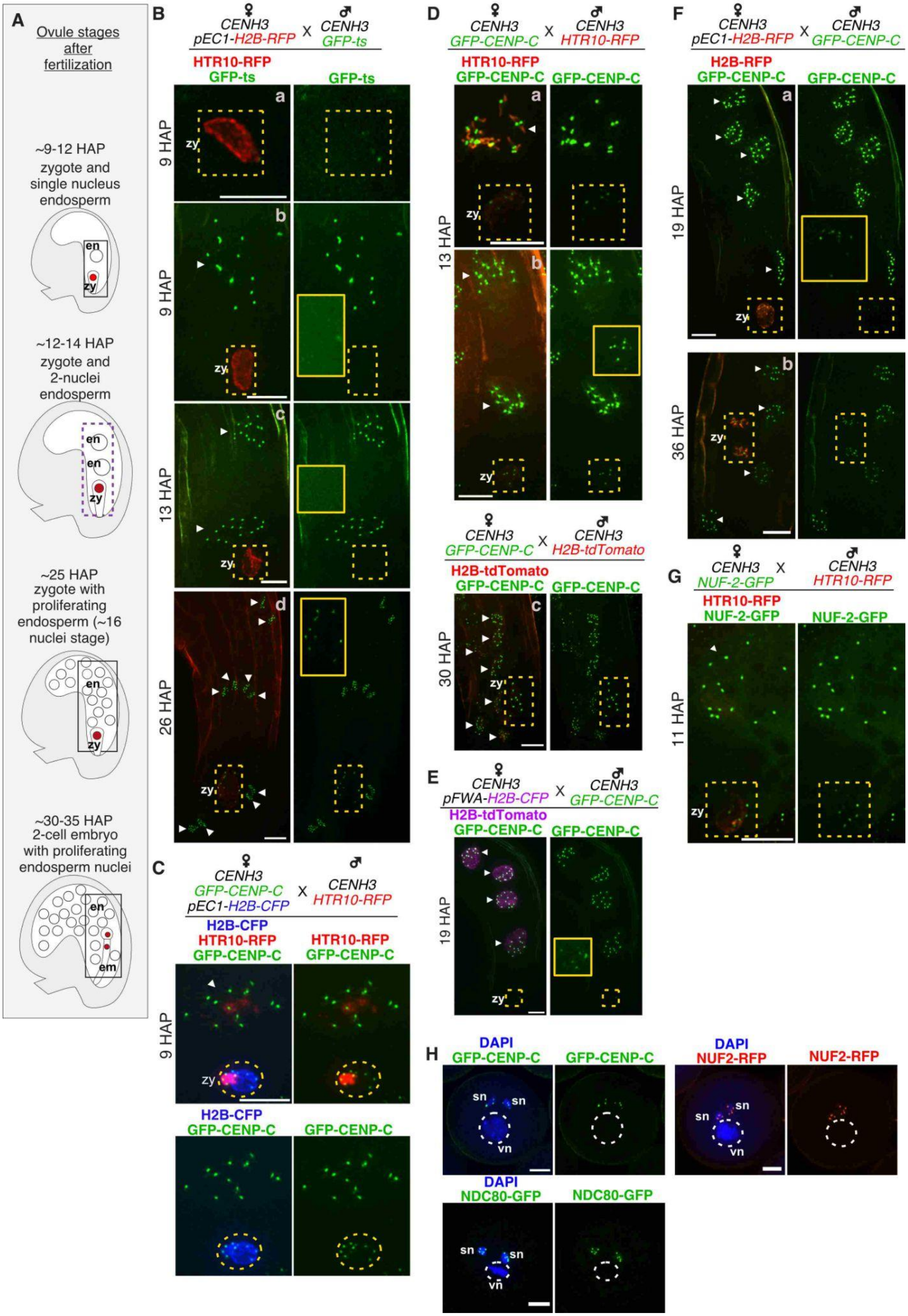
A
Ovule stages after fertilization
~9-12 HAP zygote and single nucleus endosperm
~12-14 HAP zygote and 2-nuclei endosperm
~25 HAP zygote with proliferating endosperm (~16 nuclei stage)
~30-35 HAP 2-cell embryo with proliferating endosperm nuclei
en
zy
em
B
♀ CENH3 pEC1-H2B-RFP X ♂ CENH3 GFP-ts
HTR10-RFP GFP-ts
GFP-ts
9 HAP
13 HAP
26 HAP
C
♀ CENH3 GFP-CENP-C pEC1-H2B-CFP X ♂ CENH3 HTR10-RFP
H2B-CFP HTR10-RFP GFP-CENP-C
HTR10-RFP GFP-CENP-C
H2B-CFP GFP-CENP-C
GFP-CENP-C
D
♀ CENH3 GFP-CENP-C X ♂ CENH3 HTR10-RFP
HTR10-RFP GFP-CENP-C
GFP-CENP-C
13 HAP
♀ CENH3 GFP-CENP-C X ♂ CENH3 H2B-tdTomato
H2B-tdTomato GFP-CENP-C
30 HAP
E
♀ CENH3 pFWA-H2B-CFP X ♂ CENH3 GFP-CENP-C
H2B-tdTomato GFP-CENP-C
19 HAP
F
♀ CENH3 pEC1-H2B-RFP X ♂ CENH3 GFP-CENP-C
H2B-RFP GFP-CENP-C
19 HAP
36 HAP
G
♀ CENH3 NUF-2-GFP X ♂ CENH3 HTR10-RFP
HTR10-RFP NUF-2-GFP
NUF-2-GFP
11 HAP
H
DAPI GFP-CENP-C
GFP-CENP-C
DAPI NUF2-RFP
NUF2-RFP
DAPI NDC80-GFP
NDC80-GFP
sn
vn

Fig. S6. Loading dynamics of GFP-ts, and kinetochore proteins in embryo, endosperm and pollen.

(**A**) Schematic representation of ovules in various stages of zygotic and endosperm development highlighting the region of interest as shown in images **B** to **G**. (**B-a to B-d**) Dynamics of *CENH3;GFP-ts* as male parent in a non-GEC which generates diploid progeny. After karyogamy, paternal GFP-ts is barely visible (**B-a**) and not detected in the early stages of the zygote (**B-b and B-c**) Note that in these ovules paternal GFP-ts is loaded on to all 15 parental centromeres in 1-nucleus (**B-b**) or 2-nucleus (**B-c**) endosperm. However, paternal GFP-ts is reloaded on all 10 parental centromeres before zygotic mitosis (**B-d**) and the GFP-t also marks all centromeres in the endosperm nuclei. (**C**) Loading of maternal GFP-CENP-C on paternal centromeres immediately after fertilization in both zygote (marked by maternal H2B-CFP and paternal HTR10-RFP) and endosperm (marked by paternal HTR10-RFP). Persistence of maternal GFP-CENP-C (**D**) and paternal GFP-CENP-C (**E** and **F**) in early stages of zygote and endosperm development. (**G**) Loading of maternal NUF-2-GFP on the paternal centromeres. The yellow dotted box or circle highlights the zygote. The same area is enlarged and enhanced in the inset with a solid yellow border in **B**,**D**,**E** and **F**. White solid triangle (**B to G**) marks the endosperm nucleus. Similarly, (**H**) kinetochore proteins are absent in the vegetative nuclei of mature pollen (white dotted circles). Individual pollen grains are demarcated by autofluorescence from the pollen wall. en: endosperm; zy: zygote; em: embryo; sn: sperm nucleus; vn: vegetative nucleus; HAP: hours after pollination. Scale bars = 5μm.

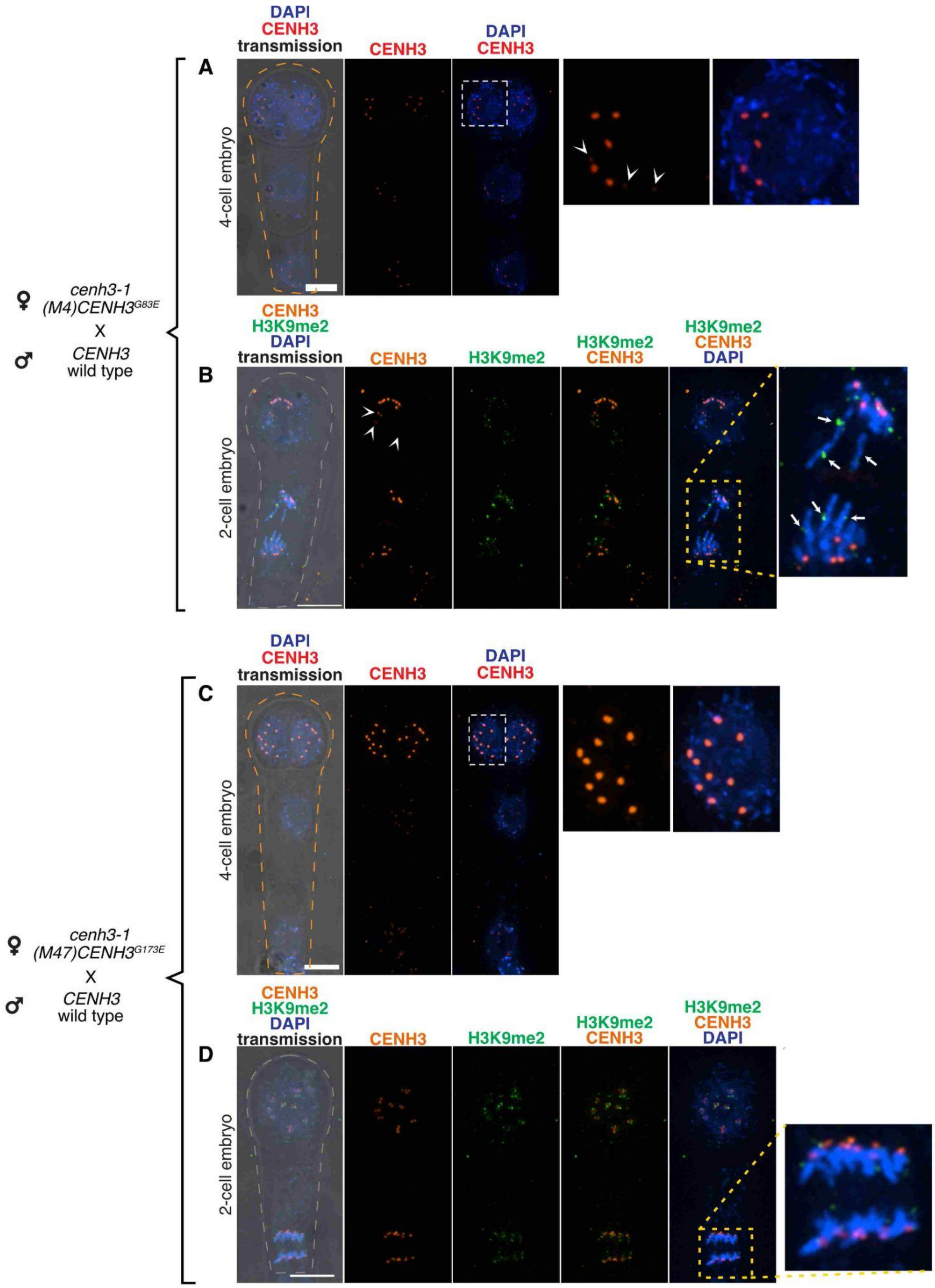

DAPI
CENH3
transmission
CENH3
DAPI
CENH3
A
4-cell embryo
cenh3-1
(M4)CENH3G83E
X
CENH3
wild type
CENH3
H3K9me2
DAPI
transmission
CENH3
H3K9me2
H3K9me2
CENH3
H3K9me2
CENH3
DAPI
B
2-cell embryo
DAPI
CENH3
transmission
CENH3
DAPI
CENH3
C
4-cell embryo
cenh3-1
(M47)CENH3G173E
X
CENH3
wild type
CENH3
H3K9me2
DAPI
transmission
CENH3
H3K9me2
H3K9me2
CENH3
H3K9me2
CENH3
DAPI
D
2-cell embryo

Fig. S7. CENH3 marks one parental set of centromeres in GE cross involving point mutations in CENH3.

(**A,B**) Biased (uniparental) localization of CENH3 on one parental centromere in GE cross (*CENH3*^{G83E} X Wild-type). White arrowheads mark fainter CENH3 signals and white arrow mark laggard chromosomes lacking CENH3. (**C,D**) Normal localization of CENH3 on both parental sets of centromeres in a control cross (*CENH3*^{G173E} X Wild-type). Marking centromeric chromatin, H3K9me2 detected on all 10 parental centromeres in both GE (**B**) and control cross (**D**). Scale bars= 5μm.

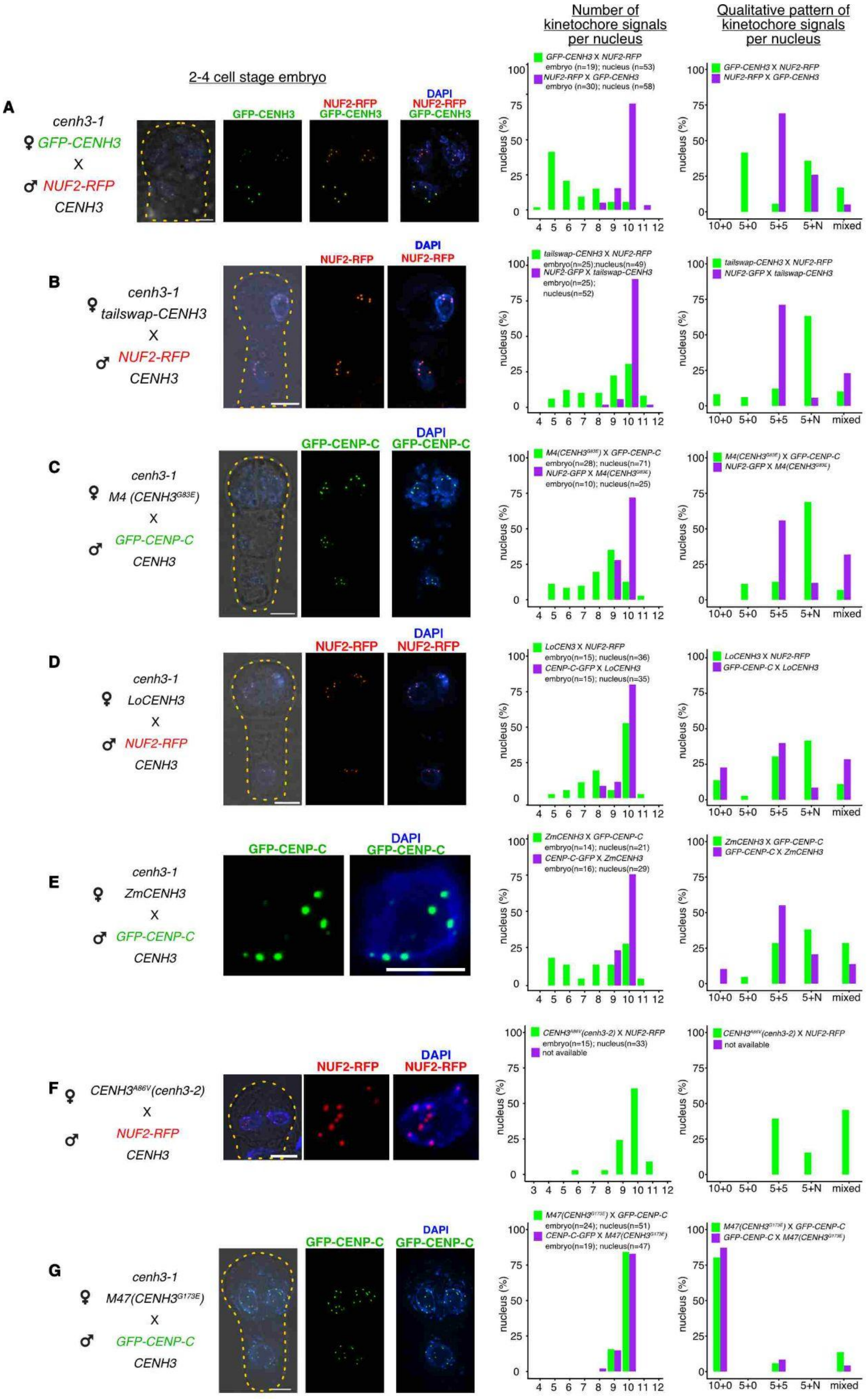

Number of kinetochore signals per nucleus
Qualitative pattern of kinetochore signals per nucleus
2-4 cell stage embryo
A
cenh3-1
♀ GFP-CENH3
X
♂ NUF2-RFP
CENH3
GFP-CENH3
NUF2-RFP GFP-CENH3
DAPI NUF2-RFP GFP-CENH3
GFP-CENH3 X NUF2-RFP embryo (n=19); nucleus (n=53)
NUF2-RFP X GFP-CENH3 embryo (n=30); nucleus (n=58)
B
cenh3-1
♀ tailswap-CENH3
X
♂ NUF2-RFP
CENH3
NUF2-RFP
DAPI NUF2-RFP
tailswap-CENH3 X NUF2-RFP embryo(n=25);nucleus(n=49)
NUF2-GFP X tailswap-CENH3 embryo(n=25); nucleus(n=52)
C
cenh3-1
♀ M4 (CENH3G83E)
X
♂ GFP-CENP-C
CENH3
GFP-CENP-C
DAPI GFP-CENP-C
M4(CENH3G83E) X GFP-CENP-C embryo(n=28); nucleus(n=71)
NUF2-GFP X M4(CENH3G83E) embryo(n=10); nucleus(n=25)
D
cenh3-1
♀ LoCENH3
X
♂ NUF2-RFP
CENH3
NUF2-RFP
DAPI NUF2-RFP
LoCEN3 X NUF2-RFP embryo(n=15); nucleus(n=36)
CENP-C-GFP X LoCENH3 embryo(n=15); nucleus(n=35)
LoCENH3 X NUF2-RFP
GFP-CENP-C X LoCENH3
E
cenh3-1
♀ ZmCENH3
X
♂ GFP-CENP-C
CENH3
GFP-CENP-C
DAPI GFP-CENP-C
ZmCENH3 X GFP-CENP-C embryo(n=14); nucleus(n=21)
CENP-C-GFP X ZmCENH3 embryo(n=16); nucleus(n=29)
GFP-CENP-C X ZmCENH3
F
♀ CENH3A86V(cenh3-2)
X
♂ NUF2-RFP
CENH3
NUF2-RFP
DAPI NUF2-RFP
CENH3A86V(cenh3-2) X NUF2-RFP embryo(n=15); nucleus(n=33)
not available
G
cenh3-1
♀ M47(CENH3G173E)
X
♂ GFP-CENP-C
CENH3
GFP-CENP-C
DAPI GFP-CENP-C
M47(CENH3G173E) X GFP-CENP-C embryo(n=24); nucleus(n=51)
CENP-C-GFP X M47(CENH3G173E) embryo(n=19); nucleus(n=47)
GFP-CENP-C X M47(CENH3G173E)
nucleus (%)
0
25
50
75
100
3 4 5 6 7 8 9 10 11 12
10+0
5+0
5+5
5+N
mixed

Fig. S8. Early stage embryos from the genome elimination cross involving different haploid inducer lines predominantly assemble five stronger kinetochores.

Two to four cell stage embryos from the genome elimination crosses (**A to F**) or control cross (**G**) involving multiple CENH3 variants. The images of embryos and nuclei are shown only for the crosses where the CENH3 variant was used as female. GFP-CENP-C or NUF2-RFP expressing lines in the *CENH3 +/+* background used as a male line. Bar graphs on the right indicate the counts of kinetochore signals, and signal pattern per nuclei of the respective genotypes shown on the left. The number of embryos and nuclei observed for each cross and used to generate bar graphs are shown in parenthesis. The “x+x” pattern indicates the number of bright + faint signal patterns within a nucleus. For eg. in the pattern 5+N, N=1,2,3,4,6. Mixed pattern indicates a nucleus with a variable number of bright signals + faint signals that do not follow the expected euploid pattern. Scale bar=5μm.

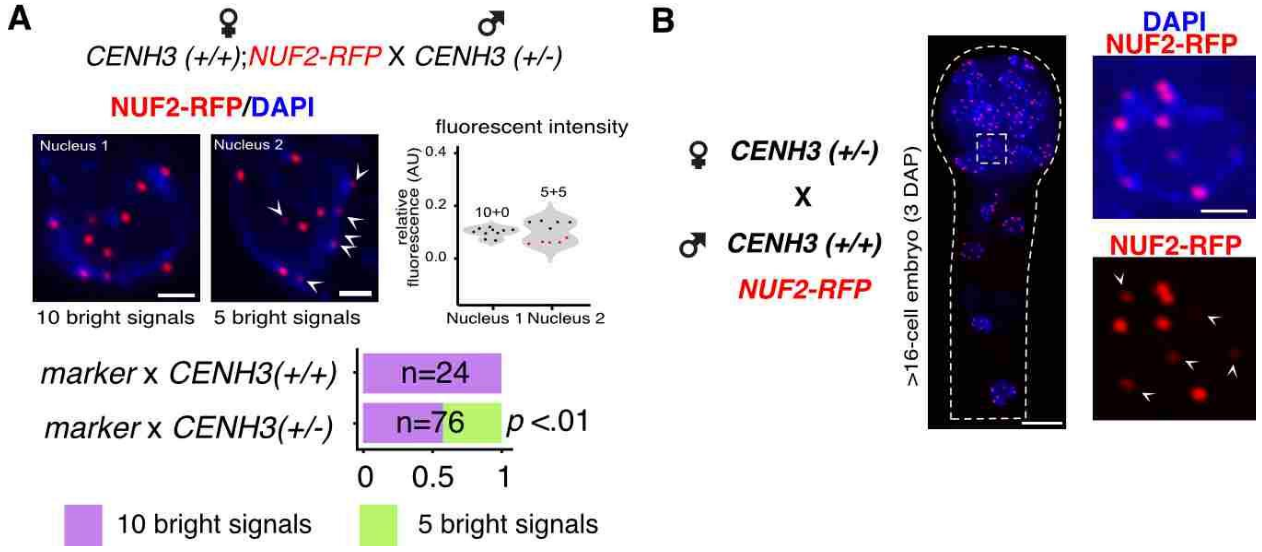

Fig. S9. Gametic dilution of CENH3 mimics GE cross.

(**A**) Patterns of kinetochore signal intensity in embryos generated by crossing *CENH3(+/-)* parent as male with line expressing kinetochore marker. White arrowheads indicate fainter kinetochore signals. Violin plot displays kinetochore signal intensity from the images on the left. Red circles in the violin plot represent faint signals in the nucleus 2. Bottom, counts for embryos analyzed in **A**. (**B**) Persistence of bright and faint kinetochore patterns in >16 cell stage embryos. A representative nucleus (white dotted box) in **B** is enlarged and shown on the right. n: number of embryos observed. Scale bar = 1μm for single nucleus shown in **A** and **B** and 5μm for embryo image in **B**.
